# Acetylation Regulates ACSL4 Degradation Through Chaperone‐Mediated Autophagy to Alleviate Intervertebral Disc Degeneration

**DOI:** 10.1002/advs.202516015

**Published:** 2025-11-21

**Authors:** Zhouwei Wu, Zhichen Jiang, Chenglong Hong, Shu Yang, Shuqing Jin, Chenyu Wu, Kaijie Guo, Jiang Liu, Shaobo Xu, Chenggui Wang, Xiangyang Wang

**Affiliations:** ^1^ Department of Orthopaedics The Second Affiliated Hospital and Yuying Children's Hospital of Wenzhou Medical University Wenzhou 325027 China; ^2^ Key Laboratory of Orthopaedics of Zhejiang Province Wenzhou 325027 China; ^3^ Zhejiang Engineering Research Center for Innovation and Application of Intelligent Prevention and Treatment of Scoliosis in Children and Adolescents Wenzhou 325027 China; ^4^ Zhejiang‐Hong Kong Joint Laboratory for Precision Diagnosis and Treatment of Spinal Disorders Wenzhou 325027 China

**Keywords:** acetylation, chaperone‐mediated autophagy, ferroptosis, intervertebral disc degeneration, senescence

## Abstract

Chaperone‐mediated autophagy (CMA) represents a critical lysosomal degradation pathway in the context of intervertebral disc degeneration (IVDD) associated with senescence. This study revealed a novel mechanism of CMA regulation involving targeted degradation of acyl‐CoA synthetase long‐chain family member 4 (ACSL4), which can delay nucleus pulposus cell (NPC) senescence and inhibit IVDD progression. Mechanistic investigations demonstrated that the acetyltransferase KAT2B can facilitate the acetylation of ACSL4 at lysine residues K500, K571, and K692. This post‐translational modification served as a molecular switch, significantly enhancing the affinity between ACSL4 and the CMA recognition chaperone HSPA8, thereby promoting the efficient targeting and degradation of ACSL4 via the CMA pathway. Besides, engineered exosomes are harnessed to deliver the key CMA receptor LAMP2A in an in vivo model, effectively delaying cellular senescence and significantly attenuating IVDD progression. Overall, these findings establish the crucial protective role of CMA in preventing IVDD through the degradation of ACSL4, providing novel insights for developing therapeutic strategies targeting CMA activation to alleviate disc degeneration and associated chronic pain.

## Graphical abstract

Intervertebral disc degeneration is triggered by ACSL4 accumulation‐mediated ferroptosis of nucleus pulposus cells due to CMA dysfunction. KAT2B promotes ACSL4 degradation via CMA through acetylation. AAV‐mediated LAMP2A delivery or engineered exosomes rescue nucleus pulposus cell senescence and disc degeneration. This study identifies CMA‐regulated ferroptosis suppression as a key mechanism in disc degeneration and proposes LAMP2A‐targeted therapy for clinical translation.

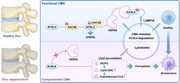

## 1 Introduction

Intervertebral disc degeneration (IVDD) is a leading cause of low back pain (LBP) in the elderly, significantly impacting physical disability, psychological distress, and socioeconomic burden.^[^ ^1^ ^]^ The degeneration of the intervertebral disc (IVD) is a complex process involving various biological and mechanical factors that contribute to the onset and progression of LBP.^[^ ^2^ ^]^ The pathophysiology of IVDD involves the degradation of the extracellular matrix (ECM), inflammation, and cellular senescence, all of which play crucial roles in the development of discogenic pain.^[^ ^3^ ^]^ With demographic shifts toward an older global population, the public health burden of LBP is becoming increasingly severe. Therefore, a deeper understanding of the pathogenesis of IVDD and the exploration of novel therapeutic targets to delay its progression are of paramount importance. The IVD comprises the central nucleus pulposus (NP), the peripheral annulus fibrosus (AF), and the superior and inferior cartilaginous endplates (EP).^[^ ^4^ ^]^ These structures work synergistically to confer spinal flexibility and stability, effectively transmitting compressive loads, making the maintenance of their structural integrity critically important.^[^ ^5^ ^]^ Critically, aging NP cells (NPCs) exhibit elevated senescence‐associated GLB1/β‐galactosidase (SA‐GLB1/β‐gal) activity and upregulated expression of senescence markers (such as TP53/p53, CDKN1A/p21, and CDKN2A/p16), accompanied by a senescence‐associated secretory phenotype (SASP).^[^ ^6^, ^7^, ^8^ ^]^ SASP factors, including IL‐1β, propagate inflammatory cascades and matrix degradation, accelerating IVDD progression.^[^ ^9^, ^10^ ^]^

Chaperone‐mediated autophagy (CMA) is a selective lysosomal degradation pathway essential for maintaining proteostasis.^[^ ^11^, ^12^ ^]^ Unlike bulk autophagy, the specificity of CMA stems from its ability to recognize and target substrate proteins containing a KFERQ‐like motif. Importantly, HSPA8 recognizes this specific motif and delivers the substrate protein to the lysosomal surface. Subsequently, the substrate is translocated into the lysosomal lumen for degradation via LAMP2A.^[^ ^13^, ^14^ ^]^ LAMP2A expression acts as the rate‐limiting factor for CMA, as it directly determines the overall flux of the pathway.^[^ ^15^ ^]^ CMA plays a crucial role in preventing the accumulation of aggregation‐prone proteins (such as α‐synuclein and tau) and in regulating physiological processes like calcium homeostasis and lipid metabolism.^[^ ^16^, ^17^, ^18^, ^19^, ^20^ ^]^ The functional decline of autophagic pathways is a hallmark of aging and a known driver of cellular senescence.^[^ ^21^ ^]^ While macroautophagy dysfunction has been extensively linked to senescence through mechanisms like impaired mitochondrial clearance and accumulation of damaged organelles, the role of the more selective CMA is equally critical but less explored. A significant decline in CMA function is observed across multiple tissues with age, strongly correlated with the senescent phenotype.^[^ ^22^ ^]^ This functional deterioration primarily arises from reduced LAMP2A transcription, decreased stability of LAMP2A at the lysosomal membrane, and a reduction in the abundance of lysosomes capable of performing CMA.^[^ ^23^ ^]^ However, the specific signaling pathways through which CMA deficiency leads to cellular senescence remain unclear.

Post‐translational modifications (PTMs), such as acetylation and ubiquitination, play vital roles in regulating gene expression and protein function.^[^ ^24^, ^25^ ^]^ Acetylation, which involves the addition of acetyl groups to lysine residues, regulates protein stability, interactions, and activity. Histone acetyltransferases (HATs) and histone deacetylases (HDACs) are now understood to modulate non‐histone protein (including transcription factors) acetylation and deacetylation, respectively, thereby controlling their stability and transcriptional activity.^[^ ^26^ ^]^ Acetylation has emerged as a key regulatory mechanism for autophagy pathways, including CMA.^[^ ^27^ ^]^ For instance, the acetyltransferase EP300 can acetylate core autophagy proteins (e.g., Beclin‐1), thereby inhibiting autophagic flux.^[^ ^28^ ^]^ Conversely, deacetylation by enzymes such as SIRT1 can activate autophagy.^[^ ^29^, ^30^, ^31^ ^]^ Although direct studies on the acetylation of specific CMA components like LAMP2A or HSPA8 are currently limited, PTMs can generate the KFERQ targeting motif required for CMA recognition on some substrate proteins, thereby expanding the pool of potential CMA substrates.^[^ ^32^, ^33^, ^34^, ^35^ ^]^ In some instances, a lysine (K) residue can functionally substitute for a glutamine (Q) residue in the motif, which, upon acetylation, acquires properties similar to Q. For example, acetylation of the glycolytic enzyme PKM2 by PCAF promotes its degradation via CMA.^[^ ^33^ ^]^ However, the specific role of acetylation in CMA within the context of IVDD remains unknown.

In this study, we demonstrated that downregulation of CMA function could induce ferroptosis via ACSL4 accumulation, thereby promoting IVDD. We provide compelling evidence that KAT2B‐mediated acetylation of ACSL4 enhances its binding to HSPA8, facilitating its degradation by CMA. Furthermore, supplementation with bone marrow mesenchymal stem cell‐derived exosomes transfected to express the key CMA rate‐limiting protein LAMP2A effectively mitigated IVDD progression.

## 2 Results

### 2.1 CMA Function Decreases in Degenerative and TBHP‐Stimulated Human Nucleus Pulposus Cells (HsNPCs)

**Figure 1:**
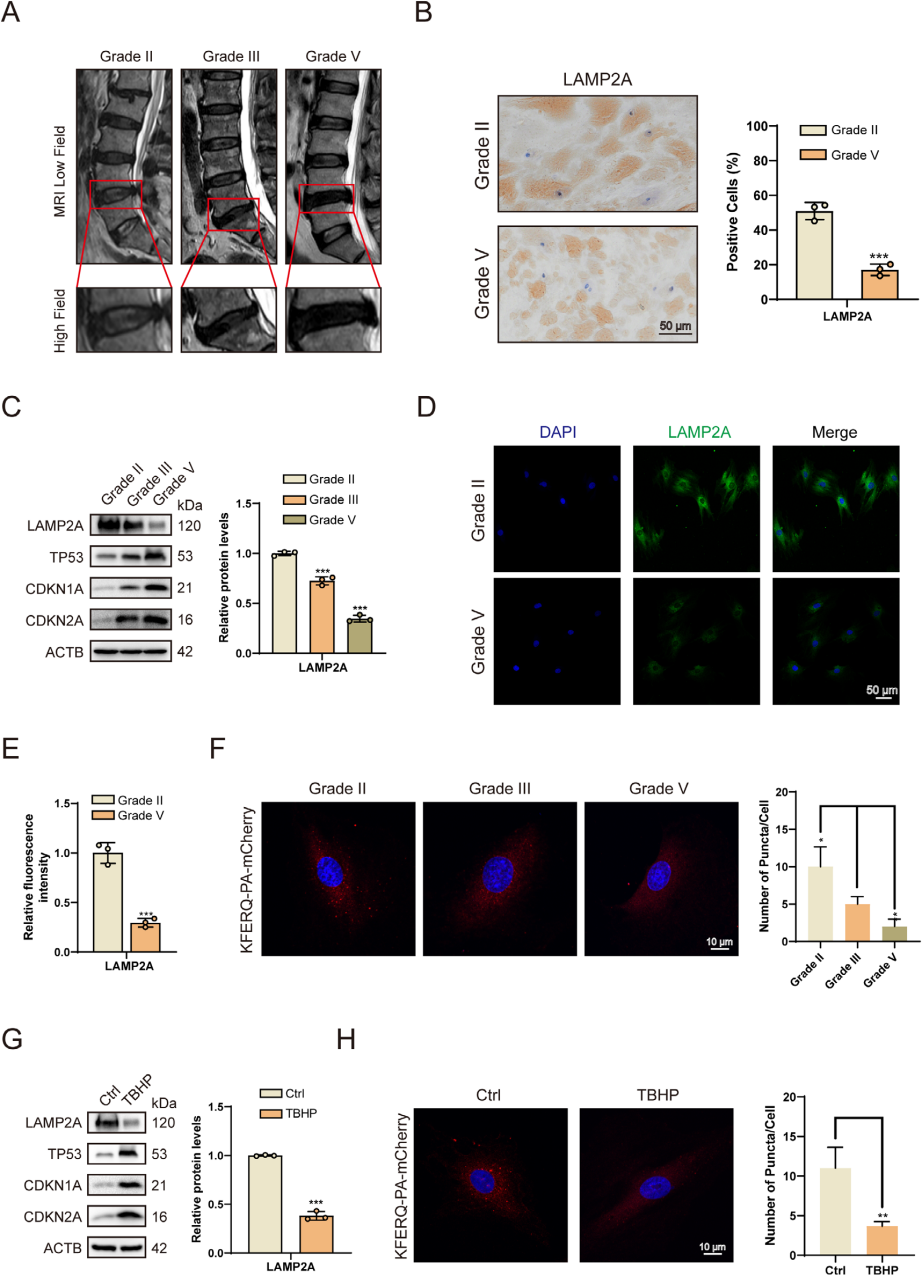
CMA function decreases in degenerative and TBHP‐stimulated HsNPCs. A) MRI in patients stratified by Pfirrmann grade for IVDD. B) IHC staining of LAMP2A in human NP tissues. (*n* = 3). ^***^p < 0.001. C) Representative western blots showing expression of LAMP2A, CDKN2A, CDKN1A, and TP53 in human NP tissues. (*n* = 3). ^***^ *p* < 0.001. D) The IF of HsNPCs confirmed the downregulation of LAMP2A expression after IVDD. (*n* = 3). E) Quantification of the mean cellular fluorescence intensity of LAMP2A in degenerated HsNPCs. (*n* = 3). ^***^ *p* < 0.001. F) Degenerated HsNPCs were transfected with the pSIN‐PAmCherry‐KFERQ‐NE reporter plasmid (red: CMA substrate; blue: DAPI). (*n* = 3). ^*^ *p* < 0.05. G) Representative western blots showing LAMP2A expression in HsNPCs decreased significantly after TBHP treatment. (*n* = 3). ^***^ *p* < 0.001. H) TBHP‐stimulated HsNPCs were transfected with the pSIN‐PAmCherry‐KFERQ‐NE reporter plasmid (red: CMA substrate; blue: DAPI). (*n* = 3). ^**^ *p* < 0.01.

CMA acts as a regulator of cellular senescence in various tissues. Given that IVDD is a progressive aging‐related disorder, we investigated the relationship between CMA and IVDD. First, patients were classified according to the Pfirrmann grading system, and NP tissue was collected from patients with varying severities of IVDD (**Figure** **1**A). We then examined the expression level of the core rate‐limiting factor of CMA, LAMP2A, in human NP tissue samples. Immunohistochemistry (IHC), qRT‐PCR, and western blot experiments showed that, compared to the control group, LAMP2A expression decreased as aging markers (CDKN1A, CDKN2A, and TP53) increased in human degenerative IVD (Figure 1B,C; Figure S1A, Supporting Information). Immunofluorescence (IF) analysis of primary NPCs extracted from human NP tissue also confirmed this finding (Figure 1D,E). To more directly assess changes in CMA function during NPC senescence, the KFERQ‐PA‐mCherry reporter system was used, given that the density of lysosome‐associated fluorescent puncta is directly correlated with CMA activity.^[^ ^36^ ^]^ We found that as NP tissue degenerated, the number of visible puncta in primary NPCs decreased, indicating that CMA activity was inhibited with IVDD progression (Figure 1F). Furthermore, given the established role of oxidative stress as an inducer of IVDD and senescence, primary HsNPCs were treated with tert‐butyl hydroperoxide (TBHP). Under these conditions, oxidative stress was observed to reduce LAMP2A expression and induce cellular senescence (Figure 1G; Figure S1B, Supporting Information). Consistent with this, the KFERQ‐PA‐mCherry reporter system revealed diminished CMA activity upon TBHP treatment (Figure 1H). Collectively, these findings demonstrated that reduced LAMP2A expression and consequent impairment of CMA function are significant features of senescent and degenerated HsNPCs and likely contribute to cellular senescence and the progression of IVDD.

### 2.2 CMA can Alleviate Senescence in HsNPCs

**Figure 2:**
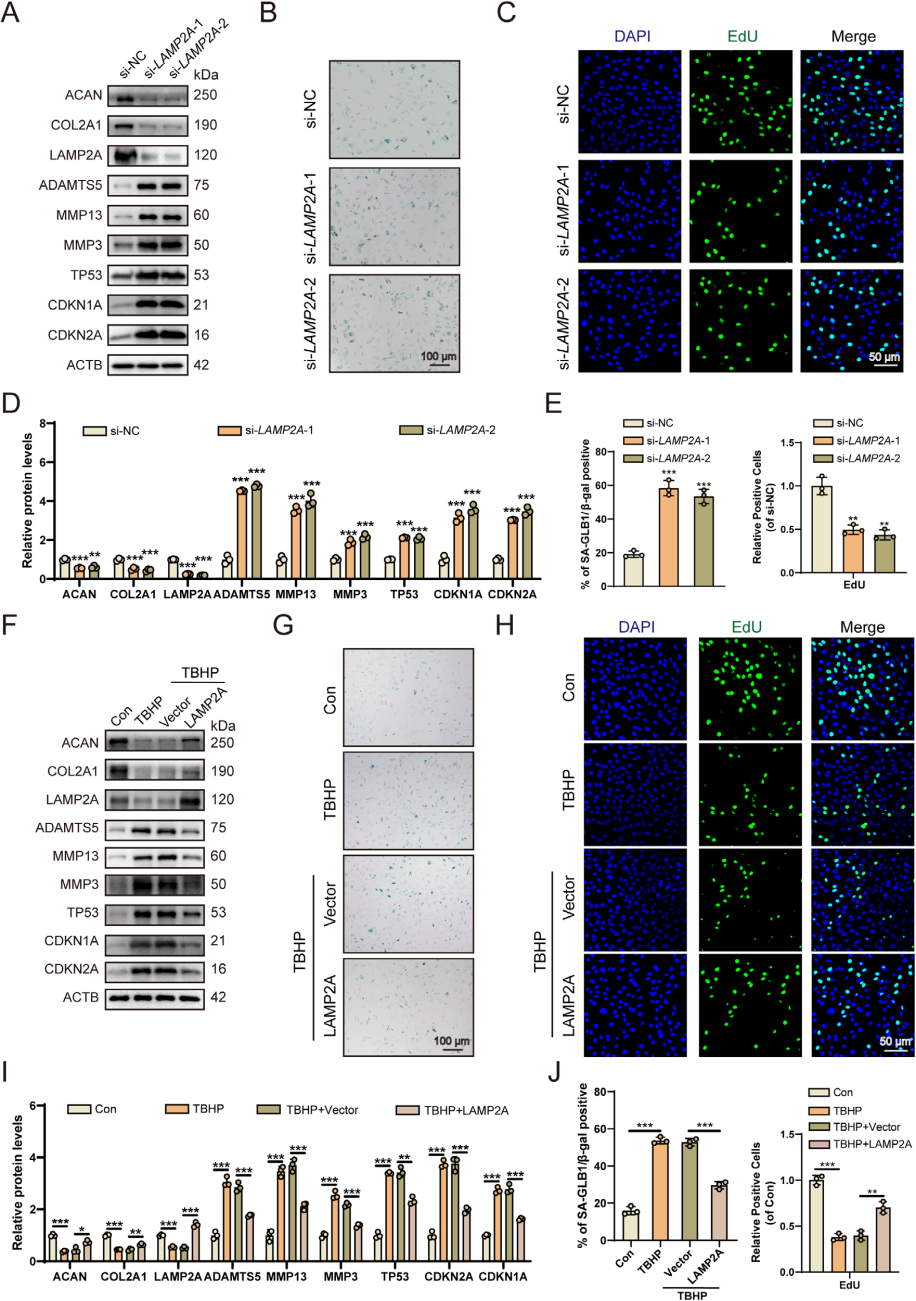
CMA can alleviate senescence in HsNPCs. A) Representative western blots showing expression of ACAN, COL2A1, LAMP2A, MMP3, MMP13, ADAMTS5, CDKN2A, CDKN1A, and TP53 in HsNPCs when *LAMP2A* was knockdown. (*n* = 3). B) Representative images of SA‐GLB1/β‐gal staining in HsNPCs when *LAMP2A* was knocked down. (*n* = 3). C) *LAMP2A* knockdown significantly suppressed HsNPCs proliferation as quantified by EdU assay. (*n* = 3). D) Quantification of the western blot in Figure 2A. (*n* = 3). ^**^ *p* < 0.01, ^***^ *p* < 0.001. E) Quantitative analysis of SA‐GLB1/β‐gal staining and EdU staining in (Figure 2B,C) (*n* = 3). ^**^ *p* < 0.01, ^***^ *p* < 0.001. F) Representative western blots showing expression of ACAN, COL2A1, LAMP2A, MMP3, MMP13, ADAMTS5, CDKN2A, CDKN1A, and TP53 in HsNPCs. (*n* = 3). G) Representative images of SA‐GLB1/β‐gal staining in HsNPCs. (*n* = 3). H) LAMP2A overexpression significantly enhanced HsNPCs proliferation in EdU assays (*n* = 3). I) Quantification of the western blot in (Figure 2F). (*n* = 3). ^*^ *p* < 0.05, ^**^ *p* < 0.01, ^***^ *p* < 0.001. J) Quantitative analysis of SA‐GLB1/β‐gal staining and EdU staining in (Figure 2G,H) (*n* = 3). ^**^ *p* < 0.01, ^***^ *p* < 0.001.

To investigate the role of LAMP2A‐mediated CMA in regulating senescence in HsNPCs, two specific small interfering RNA (siRNA) molecules targeting *LAMP2A* were designed, which reduced *LAMP2A* mRNA levels in HsNPCs in a sustained manner (Figure S2A, Supporting Information). Subsequent western blot analysis confirmed that *LAMP2A* knockdown significantly increased the expression of CDKN2A, CDKN1A, TP53, ADAMTS5, MMP3, and MMP13, while decreasing the expression of ACAN and COL2A1 (**Figure** **2**A,D). Furthermore, experiments assessing cell proliferation and senescence revealed that *LAMP2A* knockdown significantly promoted cellular senescence and inhibited cell proliferation (Figure 2B,C,E).

To further explore the therapeutic potential of LAMP2A in TBHP‐induced HsNPCs, an overexpression plasmid was constructed to upregulate *LAMP2A* mRNA expression (Figure S2B, Supporting Information). Subsequent western blot analysis demonstrated that *LAMP2A* overexpression significantly attenuated the TBHP‐induced upregulation of CDKN2A, CDKN1A, TP53, ADAMTS5, MMP3, and MMP13, while enhancing the expression of ACAN and COL2A1 (Figure 2F,I). Moreover, *LAMP2A* upregulation was associated with a decreased proportion of senescent HsNPCs and a concomitant increase in proliferative potential, as measured by SA‐GLB1/β‐gal and Edu assays. (Figure 2G,H,J). Collectively, these results indicate that LAMP2A can mitigate TBHP‐induced senescence in HsNPCs.

### 2.3 ACSL4 Represents a Potential Substrate of CMA

CMA is a selective cellular process that degrades specific substrates. To identify potential substrates involved in IVDD, *LAMP2A*‐overexpressing HsNPCs were generated, and quantitative proteomic analysis was performed to identify differentially expressed proteins following LAMP2A overexpression (Figure S3A,B, Supporting Information). We found that ACSL4 was significantly downregulated in the OE‐LAMP2A group. KEGG demonstrated a clear distinction in ferroptosis function between the control and OE‐LAMP2A clusters (Figure S3C, Supporting Information). The reduction in several known CMA substrates further validated the reliability of the proteomic results (Figure S3D, Supporting Information).

In the canonical CMA process, HSPA8 is responsible for substrate targeting and transport, while LAMP2A facilitates translocation across the lysosomal membrane. A key characteristic of CMA substrates is their interaction with both HSPA8 and LAMP2A. To verify ACSL4 as a bona fide CMA substrate, co‐immunoprecipitation (Co‐IP) assays were used to confirm the interaction of ACSL4 with both HSPA8 and LAMP2A in HsNPCs (Figure S3E, Supporting Information). IF staining revealed significant colocalization of ACSL4 with HSPA8 or LAMP2A in HsNPCs (Figure S3F,G, Supporting Information). Furthermore, knockdown of *LAMP2A* in HsNPCs promoted ferroptosis, as evidenced by increased ACSL4 and MDA levels, and decreased GPX4, FTH, and GSH. In contrast, *LAMP2A* overexpression antagonized these changes (Figure S3H–K, Supporting Information). Collectively, these findings establish ACSL4 as a potential CMA substrate implicated in IVDD pathogenesis.

### 2.4 CMA Degrades ACSL4 to Regulate its Protein Levels

**Figure 3:**
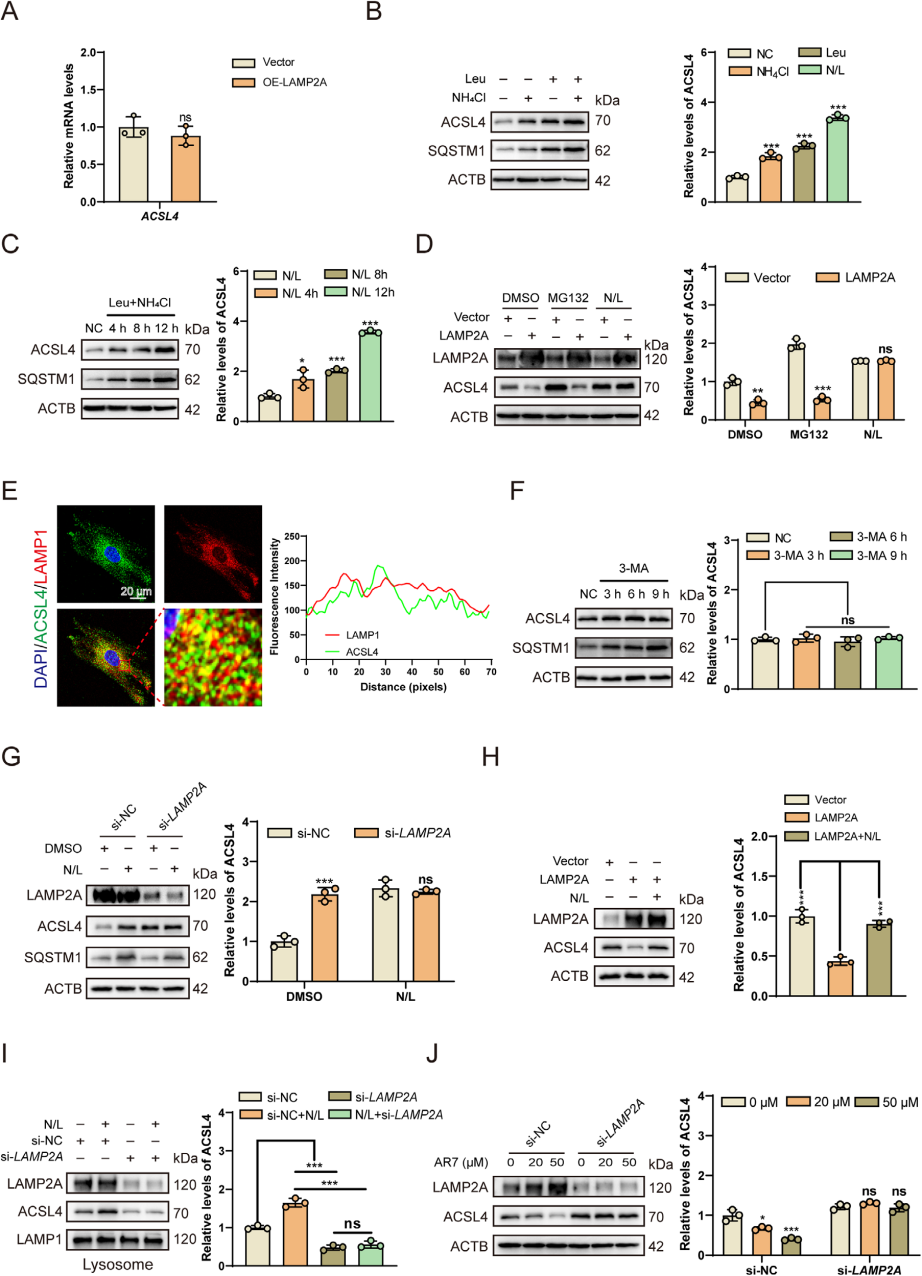
CMA degrades ACSL4 to regulate its protein levels. A) qRT‐PCR analysis indicated no mRNA level alteration of ACSL4 upon LAMP2A modulation. (*n* = 3). ns > 0.05. B) ACSL4 protein expression significantly altered in HsNPCs treated with Leupeptin (10 µm), NH_4_Cl (20 mm), or their combination for 12 h. (*n* = 3). ^***^ *p* < 0.001. C) Western blot showed ACSL4 protein levels in HsNPCs treated with a combination of Leu and NH_4_Cl (N/L) for 4, 8, and 12 h. (*n* = 3). ^*^ *p* < 0.05, ^***^ *p* < 0.001. D) Western blot analysis of ACSL4 protein in Vector and LAMP2A‐OE HsNPCs treated with MG132 and N/L for 12 h. (*n* = 3). ns > 0.05, ^**^ *p* < 0.01, ^***^ *p* < 0.001. E) The interaction between ACSL4 and LAMP1 in HsNPCs was confirmed by IF colocalization experiments. F) Western blot showed ACSL4 protein levels after 3‐MA treatment (5 mM) for 3, 6, and 9 h. (*n* = 3). ns > 0.05. G) Western blot analysis of ACSL4 expression in si‐*LAMP2A* HsNPCs treated with N/L for 12 h. (*n* = 3). ns > 0.05, ^***^ *p* < 0.001. H) Western blot analysis of ACSL4 expression in LAMP2A overexpression or combined with N/L treatment. (*n* = 3). ^***^ *p* < 0.001. I) Following 12 h exposure to N/L or vehicle, HsNPCs‐derived lysosomes were isolated for western blot analysis of ACSL4, normalized to lysosomal marker LAMP1 (n=3). ns > 0.05, ^***^ *p* < 0.001. J) Western blot analysis was performed on HsNPCs expressing si‐NC or si‐*LAMP2A* and treated with 20 µm, 50 µm AR7, or solvent. (*n* = 3). ns > 0.05, ^*^ *p* < 0.05, ^***^ *p* < 0.001.

Subsequently, we aimed to investigate and validate the pathway through which LAMP2A mediates ACSL4 degradation. qPCR analysis confirmed that *LAMP2A* overexpression did not alter *ACSL4* mRNA levels, indicating post‐translational regulation of ACSL4 protein (**Figure** **3**A). To determine whether ACSL4 undergoes lysosomal degradation, HsNPCs were treated with leupeptin (Leu), NH_4_Cl, or their combination (N/L). Elevated SQSTM1 levels confirmed effective lysosomal inhibition by these treatments (Figure 3B). Western blot analysis revealed that Leu or NH_4_Cl treatment suppressed ACSL4 degradation (Figure 3B), with these inhibitory effects being additive and time‐dependent (Figure 3B,C). Given that lysosomal pathways and the ubiquitin‐proteasome system (UPS) represent two major protein degradation mechanisms, we sought to identify the primary system regulating LAMP2A‐mediated ACSL4 stability. Our results demonstrated that combined N/L treatment, but not the proteasome inhibitor MG132, largely rescued OE‐LAMP2A‐induced ACSL4 destabilization in HsNPCs (Figure 3D), indicating that LAMP2A‐mediated ACSL4 homeostasis primarily depends on lysosomal pathways rather than UPS. Further demonstrated colocalization of ACSL4 with the lysosomal marker LAMP1 (Figure 3E). Lysosomal degradation encompasses macroautophagy, CMA, and microautophagy.^[^ ^37^, ^38^, ^39^ ^]^ To determine whether macroautophagy influences ACSL4, HsNPCs were treated with the macroautophagy inhibitor 3‐methyladenine (3‐MA). Results showed that 3‐MA did not affect ACSL4 protein levels (Figure 3F). We then investigated the necessity of LAMP2A in autophagic degradation. N/L treatment increased ACSL4 levels in si‐NC (scrambled siRNA) controls, but this effect was abolished in *LAMP2A*‐knockdown (LAMP2A‐KD) cells (Figure 3G), demonstrating that LAMP2A is required for lysosomal degradation of ACSL4. Next, LAMP2A was overexpressed in HsNPCs to enhance CMA, resulting in reduced ACSL4 levels. Co‐treatment with N/L restored ACSL4 protein accumulation (Figure 3H). To assess ACSL4 translocation to lysosomes, HsNPCs were treated with N/L. As expected, ACSL4 accumulated within the lysosomes when proteolysis was blocked. However, this accumulation was abolished in LAMP2A‐KD cells. Notably, N/L treatment failed to restore lysosomal retention of ACSL4 in LAMP2A‐KD cells (Figure 3I), indicating that LAMP2A not only facilitates ACSL4 degradation but also mediates its lysosomal translocation. Collectively, these findings demonstrate that LAMP2A‐mediated ACSL4 degradation occurs predominantly via the CMA pathway. To further validate this, we employed AR7, a specific CMA activator that does not affect macroautophagy.^[^ ^40^ ^]^ AR7 can reduce ACSL4 levels in a concentration‐dependent manner (Figure S4A, Supporting Information). Treatment with increasing AR7 concentrations failed to reverse elevated ACSL4 expression in LAMP2A‐KD HsNPCs (Figure 3J), confirming the essential role of LAMP2A in CMA‐dependent ACSL4 regulation.

### 2.5 KAT2B Mediates Acetylation of ACSL4 During IVDD

**Figure 4:**
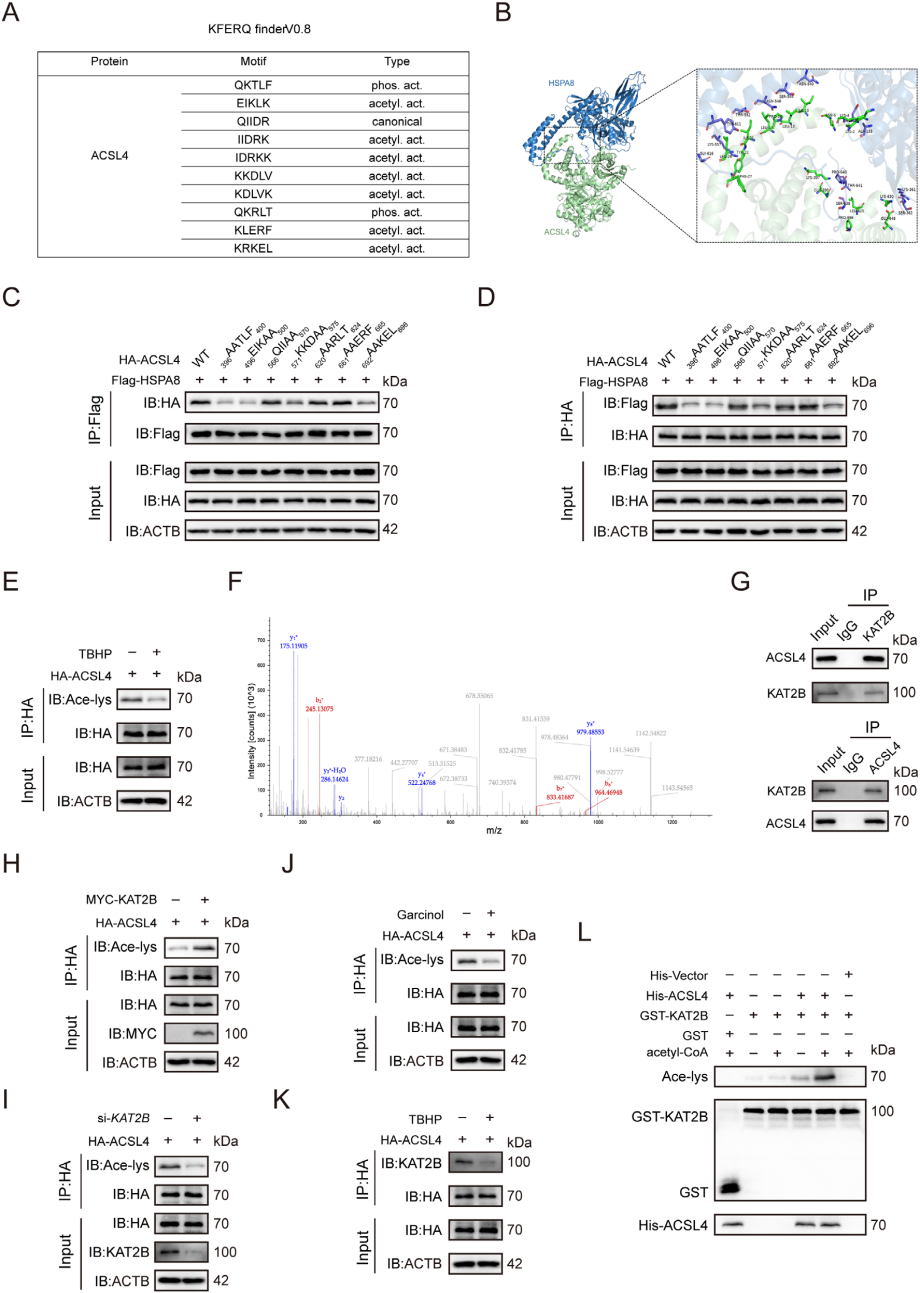
KAT2B mediates acetylation of ACSL4 during IVDD. A) The KFERQ finder software V0.8 was used to identify KFERQ‐like motifs in ACSL4. B) Molecular docking of 3D structures can predict the potential binding capacity between ACSL4 and HSPA8. C, D) Co‐IP assays confirmed the interaction between Flag‐tagged HSPA8 and HA‐tagged ACSL4 (wild‐type or mutant) in HEK293T cells. E) Acetylation of exogenously expressed HA‐ACSL4 in HsNPCs treated with TBHP was analyzed. HA‐ACSL4 was immunoprecipitated using an anti‐HA antibody, and the precipitates were probed with an anti‐acetyl‐lysine (Ace‐lys) antibody. F) The secondary spectrum of KAT2B in IP‐MS results. G) Co‐IP revealed the interaction between ACSL4 and KAT2B. H) The overexpression of *KAT2B* increases the acetylation level of ACSL4. I) Knockdown of *KAT2B* reduces the acetylation level of ACSL4. J) Chemically inhibiting KAT2B activity reduces ACSL4 acetylation. HsNPCs stably expressing HA‐ACSL4 were treated with 100 µm Garcinol, a KAT2B‐specific inhibitor, for 4 h. Cell lysates were immunoprecipitated with anti‐HA magnetic beads, followed by western blotting with the indicated antibodies. K) The treatment with TBHP impairs the binding capability of ACSL4 to KAT2B in HsNPCs. L) In vitro acetylation assay of ACSL4. Purified His‐ACSL4 was incubated with GST‐KAT2B in the reaction buffer in the presence or absence of acetyl‐CoA. ACSL4 acetylation was detected by western blotting.

Given that the CMA substrate must contain a KFERQ‐like motif, and HSPA8 plays an essential chaperone role in binding these substrates, we examined whether HSPA8 interacts with ACSL4 via specific KFERQ recognition sequences. Bioinformatics analysis using KFERQ Finder V0.8 identified multiple KFERQ‐like motifs in ACSL4 (**Figure** **4**A).^[^ ^41^ ^]^ Molecular docking further revealed a strong binding affinity between ACSL4 and HSPA8 (Figure 4B). To functionally identify critical motifs required for recognition and degradation, ACSL4 mutants were generated with double alanine substitutions in predicted CMA motifs (_396_AATLF_400_, _496_EIKAA_500_, _566_QIIAA_570_, _571_KKDAA_575_, _620_AARLT_624_, _661_AAERF_665_, _692_AAKEL_696_). Co‐IP demonstrated significantly impaired HSPA8 binding in these mutants (Figure 4C,D). Consistent with this finding, ACSL4 with the mutated motif was more resistant to AR7‐induced protein degradation compared to the wild‐type protein (Figure S4B, Supporting Information). Notably, acetylation participates in the formation of KFERQ‐like motifs in several of these sequences. Consistent with this, TBHP‐induced cellular senescence in HsNPCs progressively reduced ACSL4 acetylation levels (Figure 4E). To identify the acetyltransferase responsible for ACSL4 modification, immunoprecipitation‐mass spectrometry (IP‐MS) analysis was performed, identifying the top 15 acetylation‐related protein interactors (Table S4, Supporting Information). Among all detected acetyltransferases, KAT2B demonstrated strong composite advantages in terms of identification confidence, spectral match quality, and protein abundance, supported by a substantial number of unique peptides. Although other acetyltransferases (ACAT1/2) exhibited superior scores, they are primarily involved in cholesterol metabolism. Consequently, KAT2B was selected as the most promising ACSL4 acetyltransferase for further validation (Figure 4F). Subsequent co‐IP confirmed a specific ACSL4‐KAT2B interaction (Figure 4G). Functional validation revealed that *KAT2B* overexpression enhanced acetylation of HA‐tagged ACSL4 (Figure 4H), while *KAT2B* knockdown reduced it (Figure 4I). Pharmacological inhibition using garcinol, a KAT2B‐specific inhibitor, significantly suppressed ACSL4 acetylation in HA‐ACSL4‐expressing HsNPCs (Figure 4J). Furthermore, TBHP‐induced senescence weakened the ACSL4‐KAT2B interaction (Figure 4K). Critically, in vitro acetylation assays using recombinant KAT2B and ACSL4 purified from *E. coli* confirmed direct acetylation of ACSL4 by KAT2B (Figure 4L). These data collectively establish KAT2B as the direct acetyltransferase responsible for ACSL4 modification.

### 2.6 ACSL4 is Acetylated by KAT2B at Lysine 500, Lysine 571, and Lysine 692

**Figure 5:**
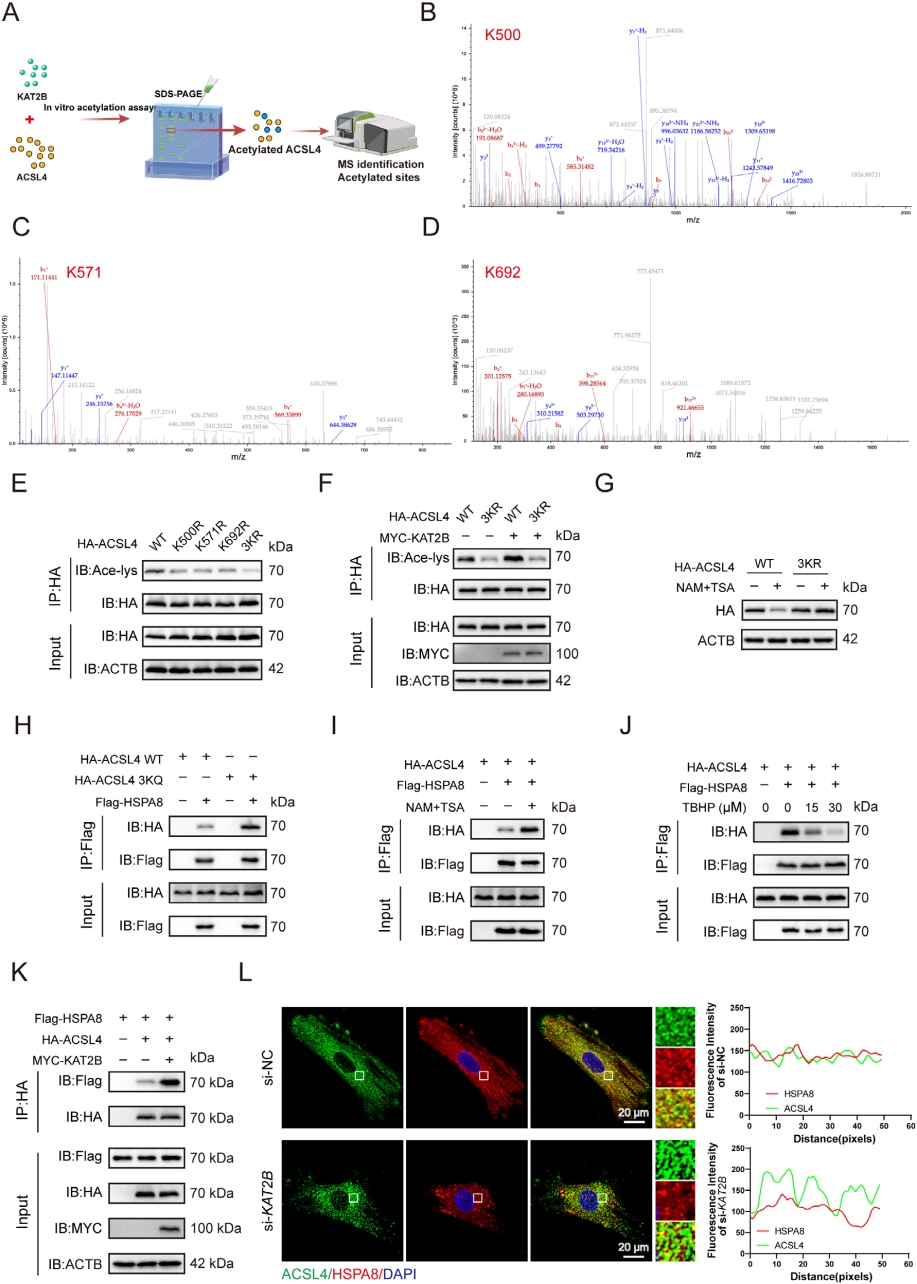
ACSL4 is acetylated by KAT2B at Lysine 500, Lysine 571, and Lysine 692. A) Schematic diagram of the in vitro acetylation assay using purified ACSL4 and KAT2B, with acetylation sites detected by mass spectrometry. B–D) Acetylation sites K500, K571, and K692 of ACSL4 were identified by mass spectrometry. E) Acetylation levels of ACSL4 mutants. 3KR represents the triple‐point mutant where Lys500, Lys571, and Lys692 residues are simultaneously substituted with arginine. F) Acetylation levels of ACSL4 mutants with or without KAT2B transfection. G) Treatment with TSA and NAM reduces wild‐type ACSL4 levels but does not affect ACSL4 mutant levels. H) The acetylation‐mimicking ACSL4 mutant enhances the binding between ACSL4 and HSPA8. I) Treatment with TSA and NAM promotes the binding between ACSL4 and HSPA8. J) TBHP‐induced cellular senescence attenuates the binding between HSPA8 and ACSL4. K) KAT2B overexpression enhances the binding between ACSL4 and HSPA8. L) IF staining demonstrates that *KAT2B* knockdown attenuates the colocalization of ACSL4 and HSPA8.

To determine whether ACSL4 acetylation sites correspond to lysine residues within its KFERQ‐like motifs, mass spectrometry analysis was performed on acetylated ACSL4 from in vitro acetylation assays (**Figure** **5**A). This analysis revealed three evolutionarily conserved acetylation sites: K500, K571, and K692 (Figure 5B–D; Figure S5A, Supporting Information), suggesting their potential as major regulatory residues. We validated these sites by transfecting HEK 293T cells with HA‐tagged ACSL4 mutants generated through site‐directed mutagenesis, where each lysine was substituted with arginine. Compared to wild‐type (WT) ACSL4, single mutants (K500R, K571R, K692R) and the triple mutant (3KR: K500R/K571R/K692R) exhibited significantly reduced acetylation (Figure 5E). Overexpression of KAT2B failed to restore acetylation in these mutants (Figure 5F), confirming KAT2B‐dependent modification at these residues. To further examine acetylation‐mediated regulation, cells stably expressing HA‐ACSL4 WT or HA‐ACSL4 3KR were treated with deacetylase inhibitors TSA and NAM. While inhibitor treatment reduced WT ACSL4 protein levels, HA‐ACSL4 3KR remained unaffected (Figure 5G), demonstrating that acetylation at K500/K571/K692 critically regulates ACSL4 stability.

The inverse correlation between ACSL4 acetylation and protein levels prompted an investigation into its role in degradation. Given that HSPA8 recruits CMA substrates to lysosomes, the binding affinity was first assessed using acetylation‐mimetic mutants in which lysines at K500, K571, and K692 were replaced with glutamines. Co‐IP revealed substantially stronger HSPA8 binding to 3KQ mutants than to WT ACSL4 (Figure 5H). Moreover, the ACSL4 3KQ mutant is more prone to degradation upon CHX treatment (Figure S5B, Supporting Information). Consistently, TSA/NAM treatment enhanced ACSL4‐HSPA8 interaction (Figure 5I). Conversely, TBHP‐induced senescence progressively weakened this binding (Figure 5J). Critically, *KAT2B* overexpression strengthened ACSL4‐HSPA8 binding (Figure 5K), while *KAT2B* knockdown attenuated their colocalization, a finding validated by IF (Figure 5L). Subsequently, we performed siRNA‐mediated knockdown of *KAT2B*. Compared with the negative control, *KAT2B* knockdown enhanced the stability of ACSL4. Conversely, *KAT2B* overexpression promoted the degradation of ACSL4 (Figure S5C,D, Supporting Information). These data establish that KAT2B‐mediated acetylation at K500/K571/K692 enhances HSPA8 binding, thereby facilitating CMA‐dependent degradation of ACSL4.

### 2.7 ACSL4 Accumulation Triggers Ferroptosis‐Induced HsNPC Senescence

ACSL4 has been established as a critical regulator of ferroptosis.^[^ ^42^ ^]^ We examined ACSL4 expression across different grades of degeneration. The results demonstrated that as disc degeneration progressed, the expression of ACSL4 and the accumulation of MDA exhibited a progressive increase. Conversely, there was a concomitant decrease in the expression of GPX4 and FTH, along with a reduction in GSH levels (Figure S6A,B, Supporting Information). Subsequently, we induced senescence in HsNPCs using TBHP. This treatment recapitulated the aforementioned phenotype, evidenced by upregulation of ACSL4, accumulation of MDA, and decreased levels of GPX4, FTH, and GSH (Figure S6C,D, Supporting Information). Taken together, these findings demonstrate that ferroptosis is elevated during the progression of IVDD.

As a specific CMA substrate, the accumulation of ACSL4 under CMA‐deficient conditions can contribute to intracellular lipid peroxidation and enhance cellular susceptibility to ferroptosis, thereby inducing cellular senescence. To confirm the relationship between ACSL4 and cellular senescence, two *ACSL4*‐targeting siRNAs were designed, and their knockdown efficiency was validated (Figure S6E, Supporting Information). Subsequent western blot demonstrated that *ACSL4* knockdown reduced expression of senescence markers (CDKN1A, CDKN2A, TP53) and matrix‐degrading enzymes (MMP3, MMP13, ADAMTS5), while enhancing ECM components (ACAN, COL2A1) (Figures S7A and S6F, Supporting Information). SA‐GLB1/β‐gal staining and EdU assays revealed decreased abundance of senescent HsNPCs and enhanced proliferative capacity upon ACSL4 depletion (Figures S7B,C and S6H, Supporting Information). Moreover, knockdown of *ACSL4* significantly suppressed ferroptosis (Figure S6G,I, Supporting Information). Consistently, BODIPY™ 581/591 C11, FerroOrange, and ROS probes showed reduced lipid peroxidation, Fe^2^⁺ accumulation, and ROS levels (Figure S7D–F, Supporting Information). To further substantiate the contribution of ACSL4‐driven ferroptosis to the senescence process, a rescue experiment was conducted utilizing a specific ferroptosis inhibitor in HsNPCs overexpressing *ACSL4*. The results demonstrated that pharmacological inhibition of ferroptosis effectively suppressed the hallmarks of ferroptosis, confirming its critical role in this model (Figure S8A,C–F, Supporting Information). Notably, this intervention successfully rescued the ACSL4‐induced degenerative phenotypes, including the excessive degradation of the ECM and the onset of cellular senescence (Figure S8A,B, Supporting Information). These results indicate that ACSL4 accumulation induces ferroptosis, ultimately driving HsNPC senescence and degeneration.

To further establish that ACSL4 and ferroptosis act downstream of CMA to mediate its effects, we performed two key experiments. First, co‐overexpression of LAMP2A and ACSL4 in HsNPCs revealed that ACSL4 overexpression effectively counteracted the beneficial effects of LAMP2A. Specifically, it reversed the anti‐senescent and pro‐proliferative effects of LAMP2A, as demonstrated by increased senescence markers, elevated SA‐GLB1/β‐gal activity, and reduced EdU incorporation (Figure S7G–I, Supporting Information). Concurrently, ACSL4 overexpression also reversed LAMP2A‐mediated suppression of ferroptosis (Figures S7J–L and S6K,L, Supporting Information). Second, and more importantly, pharmacological inhibition of ferroptosis in *LAMP2A*‐knockdown HsNPCs significantly rescued the observed phenotypes. The ferroptosis inhibitor effectively attenuated lipid peroxidation and cell death. Furthermore, it crucially mitigated both ECM degradation and cellular senescence. These protective effects were demonstrated by reduced SA‐GLB1/β‐gal activity and decreased expression of senescence markers. (Figure S8G–L, Supporting Information). Together, these genetic and pharmacological data demonstrate that CMA exerts its protective roles against senescence and ECM dysregulation by negatively regulating the ACSL4‐ferroptosis axis.

### 2.8 CMA Activation Significantly Alleviates IVDD Progression

**Figure 6:**
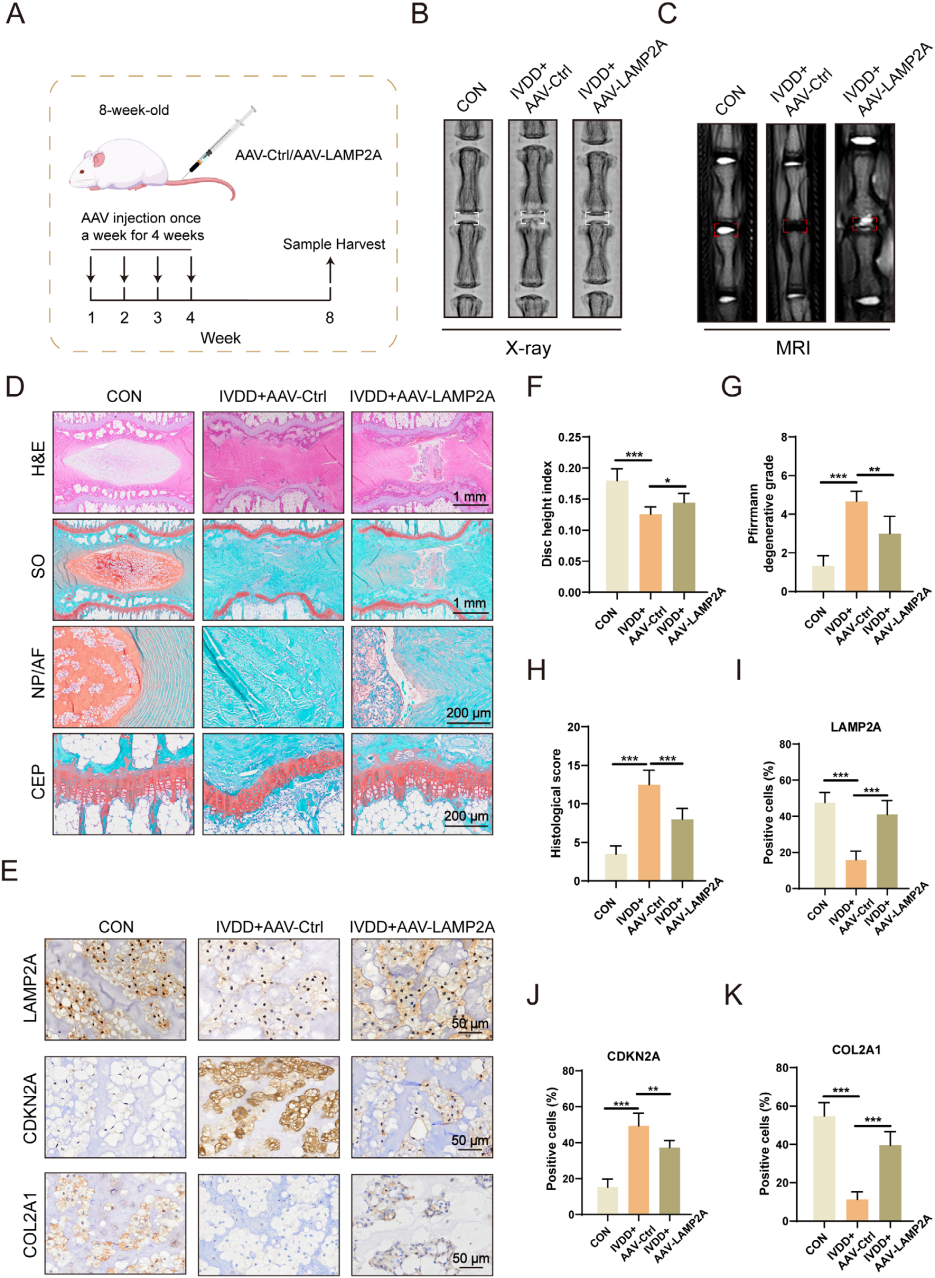
CMA activation significantly alleviates IVDD progression. A) Graphical abstract of the experimental protocol. (n = 6). B) Representative X‐ray images of rat caudal IVDs following intradiscal AAV‐Ctrl or AAV‐LAMP2A delivery. (n = 6). C) Representative MRI images of rat caudal IVDs following intradiscal AAV‐Ctrl or AAV‐LAMP2A delivery. (n = 6). D) H&E and SO staining of rat caudal IVDs. (n = 6). E) IHC staining of LAMP2A, CDKN2A, and COL2A1 in rat caudal IVDs. (n = 6). F) Disc height index (DHI). (n = 6). ^*^ *p* < 0.05, ^***^ *p* < 0.001. G) Pfirrmann degenerative grades. (n = 6). ^**^ *p* < 0.01, ^***^ *p* < 0.001. H) Histological score. (n = 6). ^***^ *p* < 0.001. I–K) Quantitative analysis of LAMP2A, CDKN2A, and COL2A1 immunostaining. (n = 6). ^***^ *p* < 0.001.

To evaluate whether CMA activation could be a potential treatment for IVDD, an AAV vector overexpressing LAMP2A was delivered into the NP of caudal IVDs in 8‐week‐old rats (**Figure** **6**A; Figure S9A, Supporting Information). Radiographic analysis demonstrated that *LAMP2A* overexpression effectively counteracted surgery‐induced disc height reduction (Figure 6B,F). Correspondingly, MRI scans revealed that LAMP2A restoration reversed IVDD‐associated hydration loss (Figure 6C,G).

Histopathological assessment using H&E and Safranin O‐Fast Green (SO) staining showed severe NP structural disorganization and AF irregularities in IVDD+AAV‐Ctrl discs. In contrast, IVDD+AAV‐LAMP2A specimens maintained greater tissue integrity with more organized annular structure (Figure 6D). The proteoglycan‐specific staining (SO) indicated significantly preserved NP tissue in LAMP2A‐treated discs. Histological scoring further confirmed LAMP2A's protective role against IVDD progression (Figure 6H). Critically, IHC revealed that treatment with AAV‐LAMP2A reduced the expression of senescence markers, increased LAMP2A and COL2A1 levels, and ultimately delayed IVDD progression (Figures 6E,I–K).

### 2.9 Engineered Exosomes Mitigate RnNPC Senescence and Suppress IVDD Progression

Building on our discovery that targeted LAMP2A activation represents a promising IVDD therapeutic strategy, we developed an engineered exosome delivery platform. Exosomes, which represent natural lipid‐bilayer nanovesicles (40–160 nm) derived from biological fluids, exhibit the ability to cross biological barriers and transport molecular cargo, making them ideal vehicles for intervertebral disc therapies.^[^ ^43^ ^]^ In the present study, exosomes were obtained from rat bone marrow‐derived mesenchymal stem cells (RnBMSCs), given their self‐renewal capacity, multilineage differentiation potential, and low immunogenicity, which contribute to their therapeutic potential.^[^ ^44^, ^45^, ^46^ ^]^

**Figure 7:**
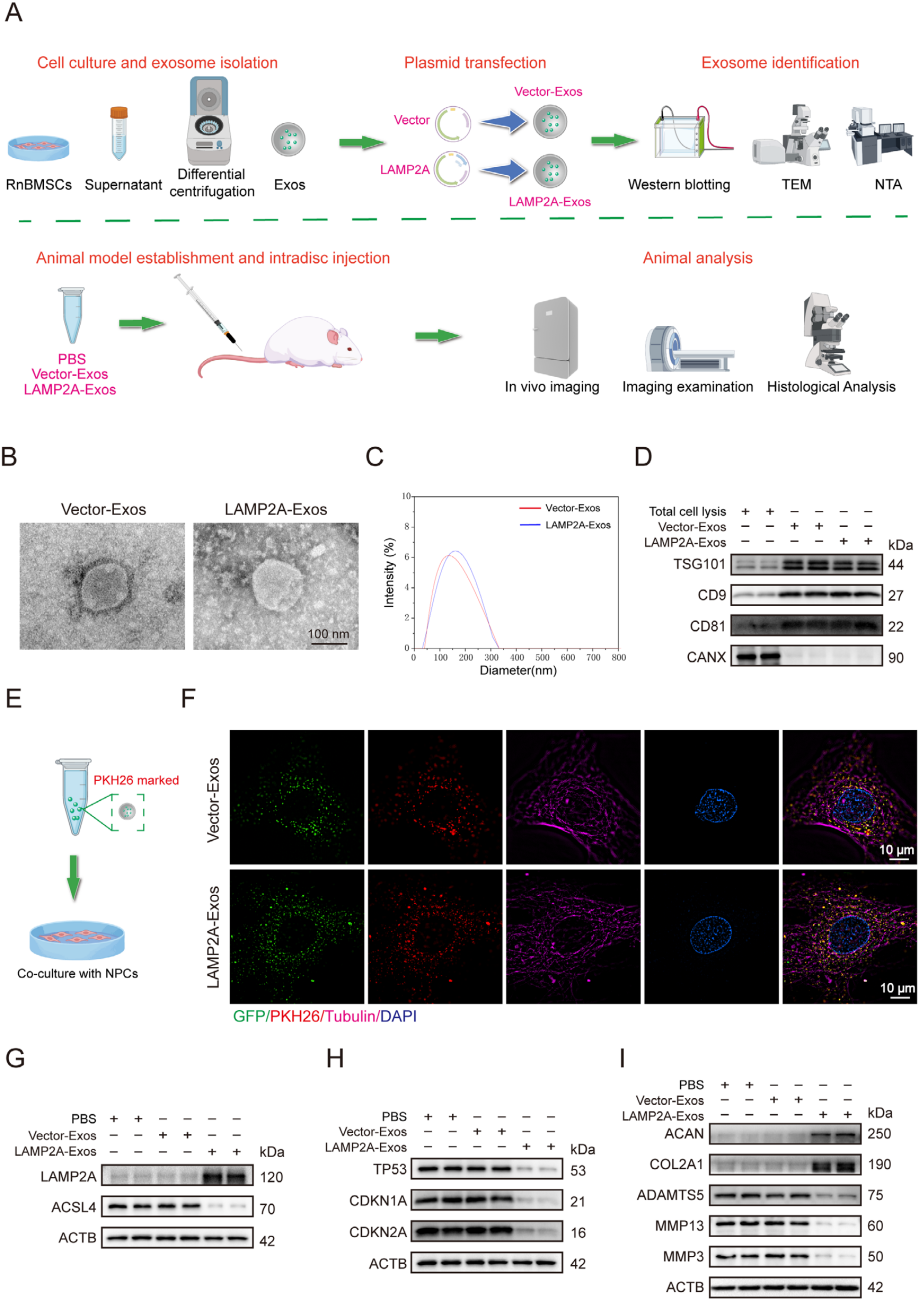
Engineered exosomes mitigate RnNPC senescence. A) Engineered exosome production and validation workflow schematic. (n = 6). B) Representative TEM images of LAMP2A‐Exos and Vector‐Exos. C) NTA quantified the size distributions of LAMP2A‐Exos and Vector‐Exos. D) Western blot analysis of exosomal biomarkers. E) Schematic diagram of co‐incubation of RnNPCs with exosomes. F) PKH26‐labeled exosomes were co‐cultured with RnNPCs for 24 h, followed by confocal microscopy visualization of cellular uptake efficiency. G, H, I) Western blot analysis of protein expression in RnNPCs after 72 h incubation with LAMP2A‐Exos or Vector‐Exos. (*n* = 3).

Exosomes purified by differential centrifugation were electroporated with LAMP2A‐overexpression plasmid or empty vector using the Exo‐Fect transfection system (**Figure** **7**A). Transmission electron microscopy and nanoparticle tracking analysis confirmed that both the LAMP2A‐Exos and VECTOR‐Exos maintained their characteristic spherical morphology with comparable size distributions (Figure 7B,C). Western blot validated the enrichment of exosomal markers (CD9, CD81, TSG101) and absence of the endoplasmic reticulum contaminant CANX (calnexin) (Figure 7D).

Notably, super‐resolution microscopy (A1‐SIM‐STORM) demonstrated significantly enhanced uptake of PKH26‐labeled LAMP2A‐Exos by RnNPCs compared to vector controls after 24 h incubation (Figure 7E,F; Figure S9B, Supporting Information). Functionally, LAMP2A‐Exos restored CMA in senescent RnNPCs by compensating for deficient LAMP2A expression. This activation effectively suppressed ferroptosis and substantially attenuated cellular senescence markers (Figure 7G–I). Collectively, these findings establish engineered exosomes as potent nanocarriers for targeted gene delivery to mitigate IVDD pathogenesis.

**Figure 8:**
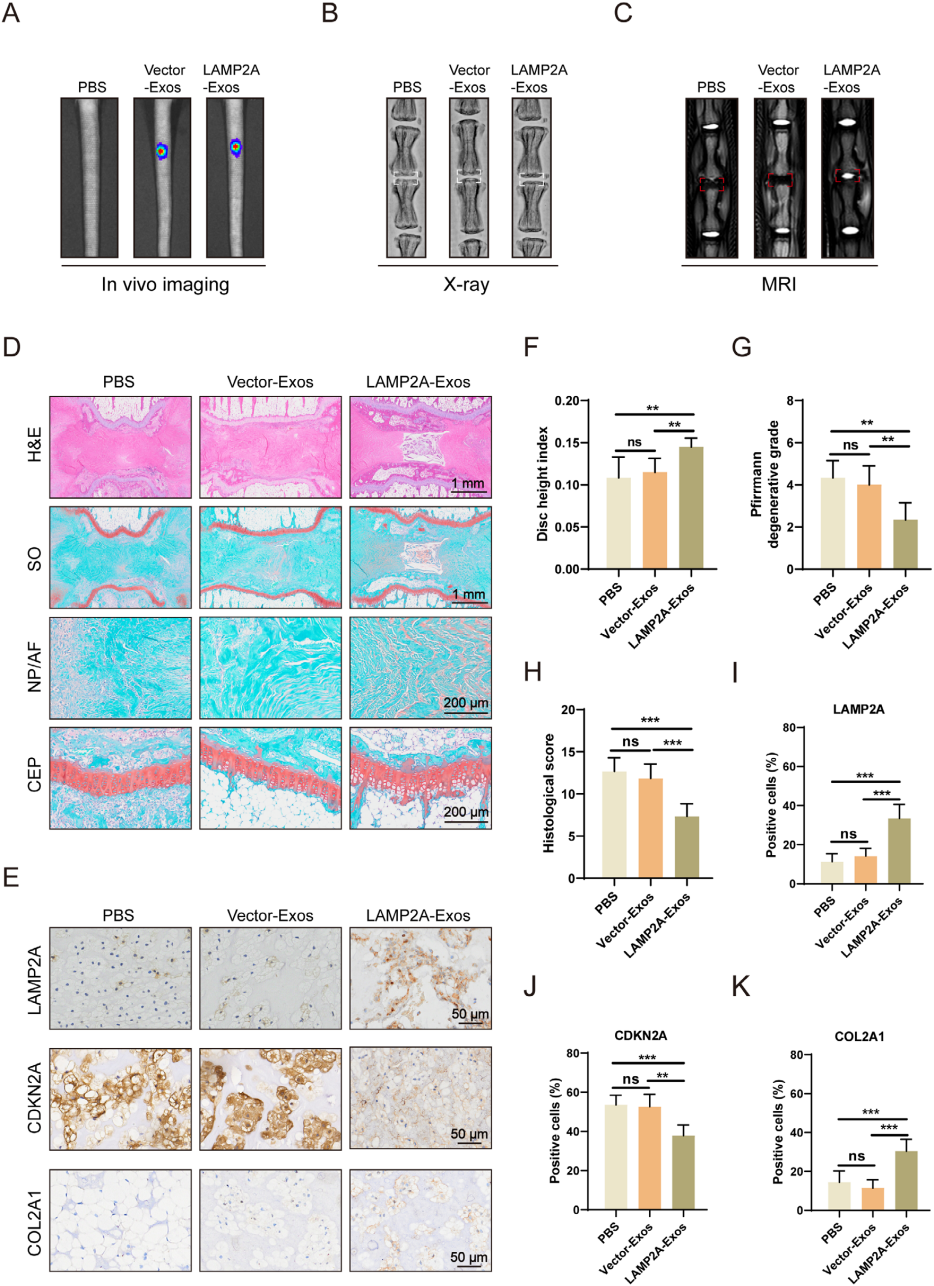
Engineered exosomes can suppress IVDD progression. A) Representative in vivo images of caudal IVDs in rats after treatment with intradiscal injection of PBS, Vector‐Exos, or LAMP2A‐Exos. B) Representative X‐ray images of rat caudal IVDs treated with intradiscal injection of PBS, Vector‐Exos, or LAMP2A‐Exos. (n = 6). C) Representative MRI images of rat caudal IVDs treated with intradiscal injection of PBS, Vector‐Exos, or LAMP2A‐Exos. (n = 6). D) H&E and SO staining of rat caudal IVDs. (n = 6). E) IHC staining of LAMP2A, CDKN2A, and COL2A1 in rat caudal IVDs. (n = 6). F) Disc height index (DHI). (n = 6). ns > 0.05, ^***^ *p* < 0.001. G) Pfirrmann degenerative grades. (n = 6). ns > 0.05, ^**^ *p* < 0.01. H) Histological score. (n = 6). ns > 0.05, ^***^ *p* < 0.001. I–K) Quantitative analysis of LAMP2A, CDKN2A, and COL2A1 immunostaining. (n = 6). ns > 0.05, ^***^ *p* < 0.001.

Subsequently, the protective effects of engineered LAMP2A‐Exos were evaluated by administering Dil‐labeled vesicles to the NP of rat caudal discs following needle puncture surgery. In vivo imaging confirmed precise localization of exosomal signals within the intervertebral space (**Figure** **8**A). Radiological assessments demonstrated that LAMP2A‐Exos treatment significantly attenuated three hallmark IVDD pathologies: disc height reduction, subchondral bone deterioration, and hydration loss (Figure 8B,C,F,G). Histological analysis further revealed LAMP2A‐Exos yielded dual therapeutic effects, downregulating senescence biomarkers while elevating expression of both LAMP2A protein and COL2A1 (Figure 8D,E,H–K). These findings demonstrate that targeted LAMP2A delivery can correct and rescue molecular deficiencies in RnNPCs, effectively delaying cellular senescence and decelerating IVDD progression. Collectively, our results establish exosome‐mediated LAMP2A overexpression as a viable therapeutic strategy for IVDD.

## 3 Discussion

The intricate relationship between IVDD and cellular senescence constitutes a critical pathological axis driving progressive, senescence‐related disc degeneration.^[^ ^47^, ^48^ ^]^ Prior research has underscored cellular senescence as a pivotal mechanistic contributor to IVDD pathogenesis, primarily through SASP factors that disrupt ECM homeostasis and propagate inflammatory cascades. This pathophysiological linkage highlights the pressing need for a comprehensive understanding of the mechanisms at the molecular, cellular, and tissue levels.^[^ ^49^, ^50^, ^51^ ^]^ Notably, advancing our understanding of senescence regulation within the disc microenvironment holds potential for therapeutic innovation. Future research directions should prioritize senescence‐targeted interventions, including senotherapeutic approaches and SASP modulation, which may provide pivotal opportunities to attenuate, halt, or even reverse IVDD progression.

CMA represents a highly selective lysosomal degradation pathway essential for maintaining cellular protein homeostasis.^[^ ^52^, ^53^ ^]^ This process selectively transports substrates into the lysosomal lumen for degradation.^[^ ^54^, ^55^ ^]^ Beyond its role in clearing misfolded or damaged proteins, CMA precisely regulates the abundance of critical functional proteins (e.g., metabolic enzymes, transcription factors), thereby participating in physiological processes such as energy metabolism, oxidative stress defense, and cell cycle regulation.^[^ ^56^, ^57^, ^58^ ^]^ Current evidence suggests that in IVDD, CMA dysfunction is closely associated with disease progression. In this respect, aging or degenerated NPCs exhibit significantly reduced LAMP2A expression, leading to impaired CMA activity. This directly results in abnormal accumulation of substrate proteins.^[^ ^17^, ^59^ ^]^ For instance, a defect in CMA prevents the degradation of phospholipase Cγ1 (PLCG1), leading to sustained activation of the IP3 pathway. This triggers a pathological overload by inducing calcium release from endoplasmic reticulum stores and influx from the extracellular environment, leading to pathological calcium overload. Calcium overload not only induces mitochondrial dysfunction and ROS bursts but also activates inflammasomes, accelerating NPC senescence and ECM degradation. Our findings confirm that CMA activity is suppressed in degenerated NP tissues. This suppression leads to the accumulation of ACSL4, a specific CMA substrate. The accumulated ACSL4 promotes lipid peroxidation, thereby inducing ferroptosis. This ferroptosis significantly exacerbates the senescence of NPCs and accelerates IVDD progression.

While KAT2B is conventionally recognized for its role in epigenetic regulation through histone acetylation,^[^ ^60^, ^61^, ^62^ ^]^ our findings reveal its novel function in post‐translationally regulation of CMA substrate selection. Contrary to its canonical epigenetic functions, KAT2B mediates acetylation at conserved lysine residues (K500, K571, K692) within ACSL4, generating a KFERQ‐like motif that enables HSPA8 recognition and subsequent lysosomal targeting. Critically, oxidative stress induced by agents such as TBHP disrupts this regulatory mechanism. Reduced KAT2B‐ACSL4 interaction compromises acetylation efficiency, impairing HSPA8 recognition and ultimately leading to pathological ACSL4 accumulation. Our work thus delineates a hitherto undocumented regulatory cascade wherein KAT2B‐mediated acetylation governs substrate‐specific CMA activation, preventing ferroptosis by eliminating a key executor of iron‐dependent cell death. This mechanism substantially expands our understanding of CMA regulation beyond transcriptional control, revealing acetylation as a rapid‐response system for substrate triage during proteotoxic stress. Furthermore, beyond this specific axis, our findings invite the broader consideration that the overall balance of CMA flux toward multiple substrates, such as ACSL4 and GPX4, could be a critical determinant in disease progression.

From a therapeutic perspective, restoring CMA activity through either AAV‐mediated LAMP2A overexpression or engineered exosomes demonstrated multifaceted efficacy in preclinical models. Both interventions effectively normalized ACSL4 turnover, suppressed ferroptotic cascades, and significantly attenuated cellular senescence markers while preserving extracellular matrix integrity. Notably, RnBMSC‐derived vesicles facilitated efficient intercellular communication via cargo delivery. Engineering these nanocarriers with LAMP2A plasmids converted them into highly internalizable “CMA reactivators”. The preservation of disc height, hydration, and proteoglycans provided compelling evidence of structurally and functionally significant CMA restoration. While these findings position CMA potentiation as a promising IVDD strategy, the contribution of additional CMA substrates to disc degeneration warrants exploration. Our findings, which demonstrate the efficacy of engineered exosomes in delivering LAMP2A to ameliorate IVDD in a preclinical model, highlight a promising therapeutic strategy. However, the translation of this approach into human clinics necessitates careful consideration of several key challenges. Future research should conduct comparative assessments of the long‐term safety profiles of viral versus exosome‐based delivery in large‐animal models and investigate the temporal dynamics of CMA impairment during IVDD progression.

**Figure 9:**
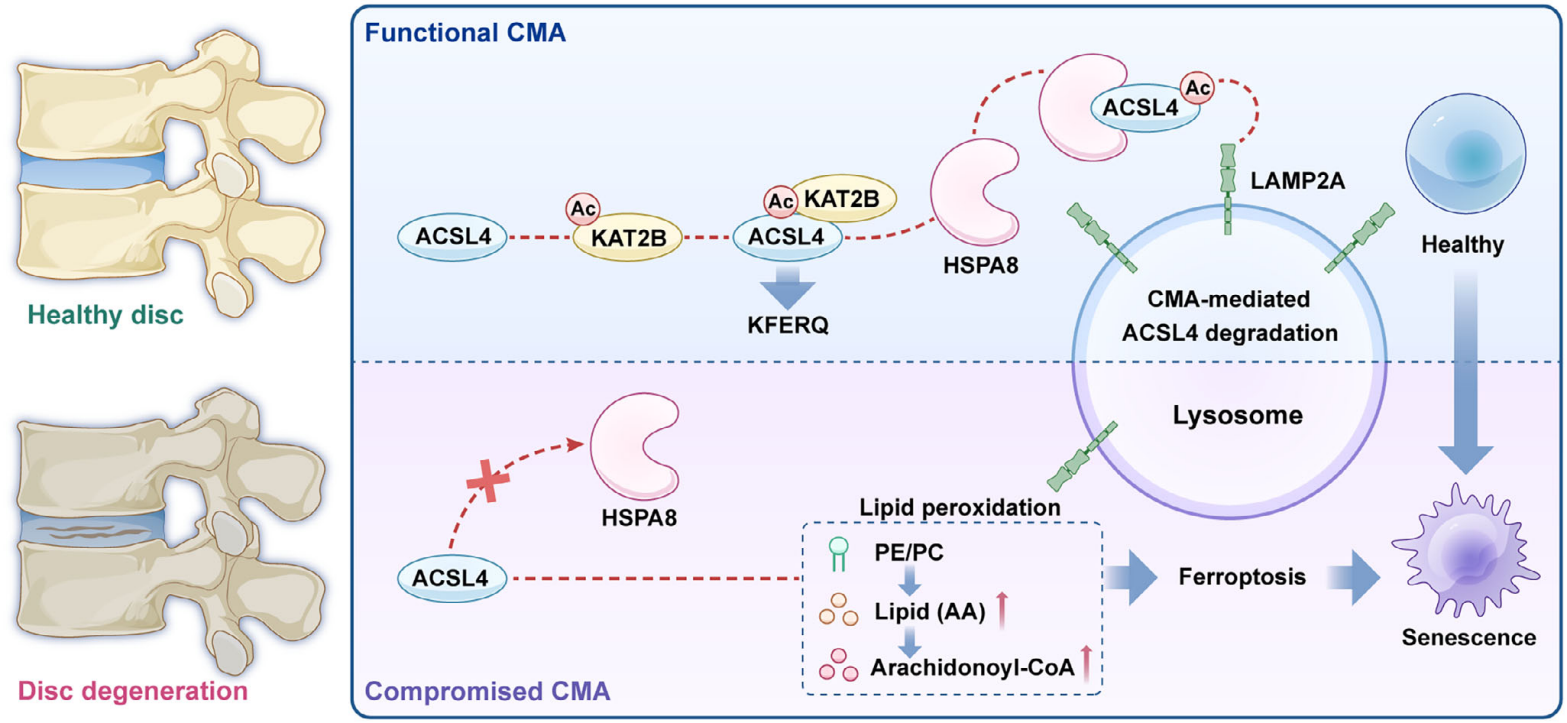
Schematic of the LAMP2A‐mediated CMA activation mechanism inhibiting ferroptosis. The activation of CMA suppresses ACSL4 accumulation‐induced ferroptosis. With the onset of NPC senescence, the binding between KAT2B and ACSL4 becomes impaired, reducing ACSL4 acetylation levels. This subsequently inhibits CMA‐dependent degradation of ACSL4, thereby exacerbating NPC senescence and accelerating IVDD progression.

Collectively, our study established impaired CMA as a pivotal mechanism driving IVDD (**Figure** **9**). We demonstrated that senescence‐related LAMP2A downregulation could compromise CMA flux in HsNPCs, leading to ACSL4 accumulation, a key ferroptosis regulator identified as a novel CMA substrate. Crucially, KAT2B‐mediated acetylation at evolutionarily conserved lysine residues (K500/K571/K692) served as a molecular switch that could target ACSL4 for CMA degradation by enhancing its interaction with the HSPA8 chaperone complex. This post‐translational regulatory axis was dysregulated during oxidative stress‐induced senescence, ultimately triggering ACSL4‐dependent iron overload, lipid peroxidation, and NPC degeneration. Overall, this work bridges fundamental autophagy mechanisms with therapeutic innovation. By elucidating how KAT2B‐mediated acetylation governs substrate‐specific CMA degradation and demonstrating the efficacy of targeted biologics, we offer a clinically relevant framework for mitigating senescence‐related disc degeneration via proteostasis restoration.

## 4 Experimental Section

### Specimen Acquisition

NP tissue samples were collected from 25 human volunteers (age range: 23–75 years, average age 48.2 years) diagnosed with lumbar vertebral fractures, idiopathic scoliosis, lumbar disc herniation, or spondylolisthesis. The degenerative status of all specimens was evaluated using the Pfirrmann grading system. This study strictly adhered to the principles outlined in the Declaration of Helsinki. Written informed consent was obtained from all participants prior to surgical procedures following committee consultation. The study protocol received formal approval from the Medical Ethics Committee of the Second Affiliated Hospital of Wenzhou Medical University (Wenzhou, China; Approval No. MR‐33‐24‐032476).

### Cell Culture

HsNPCs were isolated from Pfirrmann grade II degenerative discs. Excised tissues were minced into 1 mm^3^ fragments, washed thrice with phosphate‐buffered saline (PBS, Thermo Fisher, 10010023), and digested in 0.2% collagenase II (Thermo Fisher, 17101015) at 37 °C for 8 h. Rat NP tissues from 4‐week‐old male Sprague‐Dawley (SD) rats (n = 20) underwent identical processing with 3 h digestion. Cell suspensions were centrifuged at 300 × g for 5 min and washed twice with PBS. Pellets were resuspended in DMEM/F‐12 (Thermo Fisher, 11320033) supplemented with 10% FBS (Thermo Fisher, 10099141C) and 1% penicillin‐streptomycin (Thermo Fisher, 15140148). All cells were maintained at 37 °C/5% CO_2_.

Second‐passage HsNPCs and RnNPCs were used for experiments. HEK 293T cells (ATCC CRL‐11268) were cultured in DMEM (Thermo Fisher, 11965118) with identical supplements. Subculturing employed 0.25% trypsin (Thermo Fisher, 25200072) at 70–80% confluence.

To induce senescence, HsNPCs were treated with 30 µm TBHP (Sigma–Aldrich, 458139) for 24 h. ACSL4 protein stability was assessed following treatment with 10 µm CHX (Selleck, S7418) for 0–8 h. To activate CMA, HsNPCs were treated with AR7 (MedChemExpress, HY‐101106) at concentrations of 0, 20, or 50 µm for 24 h.

### RNA Interference and Plasmid Transfection

siRNA‐mediated knockdown was adopted to silence ACSL4, KAT2B, and LAMP2A expression in HsNPCs and HEK 293T cells. Chemically synthesized siRNAs (RIBOBIO, Guangzhou, China) were transfected using Lipofectamine 2000 (Thermo Fisher Scientific, 11668019) for 48–72 h. The siRNA sequences used in this study are provided in Table S1 (Supporting Information).

### Senescence‐Associated GLB1/β‐Galactosidase (SA‐GLB1/β‐Gal) Staining

Cellular senescence was assessed using the Senescence β‐Galactosidase staining kit (Beyotime, C0602). Following two PBS washes, cells were fixed in staining fixative for 15 min at room temperature and subsequently washed three times with PBS. Cells were then incubated overnight at 37 °C in freshly prepared staining solution without CO_2_ supplementation. Stained samples were imaged using an Olympus optical microscope.

### EdU Incorporation Assay

NPC proliferation was quantified using the BeyoClick EdU Staining Kit (Beyotime, C0071L). Cells cultured in 6‐well plates underwent pulse‐labeling with 20 µm EdU at 37 °C for 2 h. Subsequent processing included fixation in 4% paraformaldehyde for 15 min, permeabilization with 0.3% Triton X‐100 (Beyotime, P0096) for 10 min, and PBS washes. The Click reaction mixture was prepared according to the manufacturer's specifications and applied for 30 min under dark conditions. Following nuclear counterstaining with Hoechst 33342 (Beyotime, C1028) for 30 min, fluorescent images were captured using an Olympus BX53 microscope. EdU‐positive cells were quantified through ImageJ analysis.

### Western Blot

Cell lysates were prepared using ice‐cold RIPA buffer (Beyotime, P0013B) supplemented with 1% protease/phosphatase inhibitor cocktail (Beyotime, P1049) for 30 min. Following ultrasonic disruption, samples were centrifuged at 15000 × g for 30 min to collect supernatants. Protein concentrations were determined via BCA assay (Meilunbio, MA0082‐2). Samples were denatured in SDS loading buffer (Fude, FD002) at 95 °C for 10 min, separated by SDS‐PAGE, and electrotransferred to PVDF membranes. After blocking with 5% non‐fat milk for 2 h, membranes were incubated with primary antibodies overnight at 4 °C. Following three TBST washes with 0.1% Tween‐20 (Sigma–Aldrich, T9039) washes, membranes were probed with HRP‐conjugated secondary antibodies for 1 h at room temperature and washed again. Protein bands were visualized using ECL substrate (MeilunBio, MA0186‐2) and quantified with Image Lab software (v3.0, Bio‐Rad). Antibody specifications are provided in Table S2 (Supporting Information).

### Quantitative Real‐Time PCR (qRT‐PCR)

Total RNA was isolated from cells using TRIzol reagent following the manufacturer's specifications. Complementary DNA synthesis was performed with HiScript III RT SuperMix for qPCR (Vazyme, R323‐01). Quantitative real‐time PCR amplification employed ChamQ Universal SYBR qPCR Master Mix (Vazyme, Q711‐03) on a CFX96 Real‐Time PCR system (Bio‐Rad). Primer sequences for genotyping are detailed in Table S3 (Supporting Information).

### Immunofluorescence Assay

NPCs were cultured on glass coverslips and underwent specific experimental treatments prior to immunohistochemical analysis. Following treatment, cells were gently rinsed twice with PBS to remove residual media, then fixed in 4% paraformaldehyde for 15 min at room temperature to preserve cellular structures. Subsequent permeabilization was performed using 0.1% Triton X‐100 for 5 min to facilitate antibody penetration, followed by a 1 h blocking step at 37 °C with 10% goat serum (Solarbio, SL038) to minimize nonspecific antibody binding. Primary antibodies targeting proteins of interest were applied to the coverslips and incubated overnight at 4 °C to ensure thorough antigen‐antibody interaction. After two additional PBS washes to remove unbound primary antibodies, fluorophore‐conjugated secondary antibodies were added and incubated for 1 h at 37 °C to enable fluorescent detection. Nuclear counterstaining was performed using DAPI (Beyotime, C1002) for 10 min to visualize cell nuclei. Fluorescence microscopy images were acquired using an Olympus (Japan) system equipped with appropriate filters, and post‐imaging analysis, including brightness/contrast adjustments and quantitative measurements, was conducted using ImageJ software. All steps were performed under gentle agitation where necessary to ensure uniform reagent distribution, and care was taken to protect samples from light exposure during fluorescent antibody incubation and imaging.

### Quantification of CMA Activity

Assessment of CMA activity was conducted using the PA‐mCherry‐KFERQ reporter system as previously established. HsNPCs were transduced with a lentiviral vector encoding the photoactivatable pSIN‐PAmCherry‐KFERQ‐NE plasmid (Addgene #102365; deposited by Dr. Shu Leong Ho). Following transfection, cells underwent photoactivation using a 405/20 nm LED array, which maintained >90% viability post‐treatment. Activated cells were then fixed, counterstained with DAPI to visualize nuclei, and mounted for imaging. Confocal fluorescence microscopy (Olympus, Japan) was employed to capture cellular images under a 60× objective lens. Quantitative analysis of CMA activity involved enumerating red fluorescent puncta per cell across a minimum of three non‐overlapping microscopic fields. This reporter system leverages the KFERQ motif recognized by CMA machinery, where photoactivation triggers irreversible fluorescence in lysosomal compartments upon autophagic degradation, enabling precise spatial and temporal tracking of CMA dynamics. All experimental conditions were rigorously controlled to ensure reproducibility and accurate interpretation of lysosomal localization patterns.

### Animals

Eight‐week‐old SD rats (250–300 g) were maintained under standardized housing conditions at the Experimental Animal Center of Wenzhou Medical University, adhering to a 12 h light/dark cycle with ambient temperature controlled at 21 °C. For procedural intervention, a 22‐gauge needle was precisely inserted 5 mm into the Co7/8 IVD space (between the 7th and 8th caudal vertebrae) under X‐ray fluoroscopy guidance. The needle was subsequently rotated 360 degrees and maintained in position for 30 s to ensure proper placement. All experimental protocols received prior approval from the Experimental Animal Ethics Committee of Wenzhou Medical University (wydw2025‐0418).

### AAV Delivery In Vivo

Eight‐week‐old SD rats (n = 18) were randomly assigned to three groups and maintained under controlled conditions. Following anesthesia with 2% pentobarbital sodium (40 mg kg^−1^), either AAV9‐LAMP2A (1.9 × 10^12^ vg/mL) or AAV9‐Ctrl (1.7 × 10^12^ vg mL^−1^) was administered weekly into the Co7/8 NP using a 33‐gauge needle over four weeks. Imaging and histopathological evaluations were performed at two months post‐injection. The viral vectors AAV9‐LAMP2A and AAV9‐Ctrl were provided by Hanbio (Shanghai, China).

### TMT‐Based Mass Spectrometry (TMT‐MS)

Protein samples stored at −80 °C were thawed on ice and lysed in pre‐mixed lysis buffer (8 M urea, 1% protease inhibitor) via sonication. Cellular debris was removed by centrifugation (4 °C, 15 000 g, 10 min), and supernatant protein concentrations were determined using the BCA assay. Equal protein amounts were normalized with lysis buffer, precipitated with 20% TCA (4 °C, 2 h), washed thrice with pre‐cooled acetone (−20 °C), and resuspended in 100 mm TEAB. Proteins were reduced with 5 mm DTT (56 °C, 30 min), alkylated with 15 mm IAA (RT, dark, 15 min), and digested overnight with trypsin (1:50, w/w) at 37 °C. Peptides were desalted using Strata X columns, lyophilized, and quantified via Pierce™ Quantitative Peptide Assay. For TMT labeling, peptides were dissolved in 100 mm HEPES, labeled with TMT reagents (RT, 1 h), quenched with 5% hydroxylamine (15 min), pooled, and desalted. High‐pH reversed‐phase HPLC (Agilent 300Extend C18) fractionated peptides into 48 components (9–57 min), consolidated into 16 fractions, and lyophilized. LC‐MS/MS analysis utilized an Easy‐nLC 1200‐Q Exactive HFX system. Peptides were separated on a 20 cm ReproSil‐Pur C18 column (1.9 µm) with a 40 min gradient (7–22% B), ionized at 2.1 kV, and analyzed in DDA mode (MS1: 400–1200 m/z, 60k resolution; MS2: 15k resolution, HCD). For Astral DIA, samples were separated via Vanquish Neo and analyzed on an Astral mass spectrometer (380‐980 m/z, 240k resolution, 299 windows, 2 m/z isolation). Data were processed using MaxQuant (v1.6.15.0) for TMT data (FDR <1%, Trypsin/P, 2 missed cleavages) and DIA‐NN for DIA analysis (FDR <1%, Trypsin, carbamidomethyl fixed, oxidation/acetyl dynamic modifications). Uniprot databases with contaminants were used for searches. This integrated workflow enables comprehensive protein identification and quantitative comparison across samples.

### Co‐Immunoprecipitation Assay

Cellular lysates were prepared by incubating cells on ice for 30 min in NP‐40 lysis buffer (Beyotime, P0013F) supplemented with 1% protease/phosphatase inhibitor cocktail and 1 mm phenylmethylsulfonyl fluoride (PMSF; Beyotime, ST506). The lysate was clarified by centrifugation at 15 000 × g for 10 min at 4 °C. The resulting supernatant was incubated with protein A/G magnetic beads (Selleck, B23201) under constant rotation overnight at 4 °C. Immunoprecipitated complexes were recovered by boiling in 1× SDS‐PAGE loading buffer and subsequently analyzed by Western blot following established protocols. All procedures were performed under sterile conditions with rigorous temperature control to maintain protein integrity.

### Immunoprecipitation Mass Spectrometry

The immunoprecipitated eluate was resolved by SDS‐PAGE. A discrete protein band corresponding to the target of interest, measuring ≈1 mm^3^, was carefully excised from the gel matrix. The gel fragment underwent sequential processing, including decolorization to remove Coomassie stain, alkylation of reduced cysteine residues, and in‐gel enzymatic digestion using trypsin. The resultant peptide extracts were analyzed by nano‐liquid chromatography‐tandem mass spectrometry employing an EASY‐nanoLC 1200 system interfaced with an Orbitrap Exploris 480 mass spectrometer (Thermo Fisher Scientific, MA, USA). Raw spectral data were processed and interpreted using PEAKS Studio v10.6 software (Bioinformatics Solutions Inc., Waterloo, Canada) for protein identification and post‐translational modification analysis. All chromatographic and mass spectrometric parameters were optimized to ensure high‐resolution separation and sensitive detection of peptides.

### Lysosome Extraction

Lysosomal fractions were isolated from HsNPC lysates using a lysosome enrichment kit (Thermo Fisher Scientific, 89839) following the protocol. The isolation procedure incorporated differential centrifugation steps to effectively separate lysosomal vesicles from other cellular components. To validate the purity and enrichment of lysosomal preparations, protein content differences of the isolated lysosomal fraction and corresponding cytoplasmic extract were analyzed by Western blot analysis.

### Protein Purification

GST‐KAT2B was expressed in *E. coli* BL21 grown in Luria‐Bertani (LB) medium. Protein expression was induced with 0.5 mm IPTG (Sigma–Aldrich, PHG0010), and recombinant protein was purified using glutathione Sepharose 4B resin (GE Healthcare Lifesciences, 17075605). Bound protein was eluted with reduced glutathione (Beyotime, S0073). For His‐tagged ACSL4, transformed BL21 lysates were centrifuged and supernatants incubated overnight with Ni‐NTA resin (Thermo Fisher, R90115) at 4 °C. Following three wash cycles, proteins were eluted with 50 mm imidazole at 4 °C for 4 h and recovered by centrifugation.

### In Vitro Acetylation Assay

Purified HIS‐ACSL4 and GST‐KAT2B proteins were incubated with acetyl‐CoA in reaction buffer (20 mm Tris‐Cl [pH 8.0], 20% glycerol, 100 mm KCl, 1 mm DTT, 0.2 mm EDTA) at 30 °C for 2 h. Following incubation, reaction mixtures were resolved and boiled in 1 × SDS‐loading buffer. Then the reaction products were analyzed by Western blot.

### ACSL4 Acetylation Site Identification by Mass Spectrometry

Putative ACSL4 bands corresponding to in vitro acetylated products were excised from SDS‐PAGE gels and processed for mass spectrometry analysis. Gel fragments underwent sequential treatment: destaining with 50% acetonitrile in 50 mm ammonium bicarbonate (NH_4_HCO_3_), reduction of disulfide bonds using 10 mm TCEP, and alkylation of free cysteine residues with 40 mm chloroacetamide (CAA). Proteolytic digestion was performed overnight at 37 °C with 2 µg trypsin in 50 mm NH_4_HCO_3_. Peptides were extracted using 50% acetonitrile containing 0.1% formic acid and analyzed by liquid chromatography‐tandem mass spectrometry (LC‐MS/MS) on an EASY‐nLC 1200 system coupled to a Q Exactive HF‐X mass spectrometer (Thermo Fisher Scientific). Chromatographic separation utilized a 60 min linear gradient (400 nL min^−1^) with mobile phase A (0.1% formic acid in water) and mobile phase B (0.1% formic acid in acetonitrile), increasing from 2% to 100% B. Mass spectrometry acquisition alternated between full scans (Orbitrap analyzer, 60 000 resolution at m/z 200) and data‐dependent MS/MS scans of the top 20 most intense precursor ions. Precursors were fragmented using a normalized collision energy (NCE) of 27% at 15000 resolution, with a dynamic exclusion window of 20 s to minimize redundant selection. Raw data were processed using Proteome Discoverer 2.4 (Thermo Fisher Scientific) with the following parameters: sequence database search against SwissProt (version 2023_09), trypsin/P as the protease (maximum 2 missed cleavages), precursor mass tolerance of 10 ppm, fragment mass tolerance of 0.02 Da. Fixed modification was carbamidomethylation of cysteine residues, while variable modifications included acetylation (protein N‐terminus and lysine residues) and oxidation of methionine. Acetylated peptides were filtered at medium confidence (1% false discovery rate) with manual validation of representative MS/MS spectra to confirm modification localization.

### Histological Analysis

At eight weeks post‐surgery, rat tail IVD specimens were harvested and subjected to thorough rinsing in PBS. Tissue samples were fixed in 4% paraformaldehyde (PFA) at 4 °C for 48 h, followed by decalcification in 10% EDTA solution at room temperature over an 8‐week period. After sequential dehydration through graded ethanol concentrations and clearing in xylene, specimens were embedded in paraffin wax. Serial sections of 5 µm thickness were prepared using a microtome. Histological evaluation employed standard staining protocols: H&E for general morphology, and SO for proteoglycan visualization. Stained sections were examined under an Olympus VS200 light microscope equipped with digital imaging capabilities. All procedures adhered to established tissue processing protocols to ensure structural preservation and staining consistency.

### IHC Staining

IHC was performed to comparatively assess COL2A1, LAMP2A, and CDKN2A expression in human NP tissues and rat caudal IVDs. Serial 5 µm sections underwent deparaffinization in xylene and graded ethanol rehydration. Endogenous peroxidase activity was quenched with 3% H_2_O_2_ for 10 min, followed by antigen retrieval using 0.4% pepsin (Servicebio, GC305004) for 30 min at 37 °C. Non‐specific binding was blocked with 10% goat serum albumin (Zhongshanbio, ZLI‐9021) for 30 min at 37 °C. Sections were subsequently incubated with primary antibodies overnight at 4 °C and corresponding secondary antibodies. Stained sections were evaluated using an Olympus VS200 microscope.

### X‐Ray and Magnetic Resonance Imaging (MRI) Examinations

Radiographic and magnetic resonance imaging were conducted to evaluate degenerative changes in rat IVDs. Postoperative disc degeneration and calcification were quantified using an XPERT.8 X‐ray system (Kubtec). Sagittal T2‐weighted MRI was performed using a Philips Intera Achieva 3.0T scanner to assess NP signal intensity variations and structural integrity.

### Rat Bone Marrow Mesenchymal Stem Cell Isolation

RnBMSCs were isolated from bilateral femoral marrow flushes of SD rats. Following density gradient centrifugation, cells were cultured in DMEM/F12 medium supplemented with 10% FBS and 1% penicillin‐streptomycin at 37 °C/5% CO_2_.

### Exosome Isolation and Plasmid Transfection

Second‐passage RnBMSCs were cultured in DMEM/F12 containing 10% exosome‐depleted FBS. Exos were isolated from conditioned media via differential ultracentrifugation: sequential centrifugation at 300 × g for 10 min, 2000 × g for 30 min, and 10 000 × g for 30 min removed cellular debris. The clarified supernatant underwent ultracentrifugation at 100 000 × g for 70 min, followed by PBS washing and centrifuged again under the same conditions. Purified exosomes were resuspended in 100 µL PBS and stored at −80 °C.

For transfection, 5 µg LAMP2A‐overexpressing plasmid or empty vector was electroporated into 50 µg exosomes (100 µg mL^−1^) using the Exo‐Fect Transfection Kit (System Biosciences, EXFT10A‐1). After 10 min incubation at 37 °C, 30 µL ExoQuick TC reagent was added and incubated for 30 min at 4 °C. Transfected exosomes were pelleted at 20 000 × g for 3 min, resuspended in PBS, and processed for downstream applications.

### Exosome Identification

Isolated exosomes were characterized through multimodal analysis. Western blot confirmed identity and purity by detecting exosomal markers (TSG101, CD63) while excluding the endoplasmic reticulum contaminant CANX. Morphological assessment employed transmission electron microscopy (Hitachi), with nanoparticle tracking analysis quantifying size distribution. For cellular uptake studies, exosomes were labeled with PKH26 red fluorescent dye (Sigma–Aldrich, MIDI26) according to the manufacturer's protocol for subsequent fluorescence imaging.

### Cellular Uptake of Exos

NPCs were seeded in 24‐well plates (3 × 10⁴ cells well^−1^) and allowed to adhere for 24 h. PKH26‐labeled exos (20 µg well^−1^) were then introduced and incubated for 12 h. Following PBS washes, cells were counterstained with Tubulin‐Tracker Deep Red (Beyotime, C2215S) and Hoechst 33342 for 1 h at 37 °C with 5% CO_2_ to visualize cytoskeletal and nuclear architecture. Exo internalization was assessed using a Nikon A1R‐SIM‐STORM super‐resolution microscope.

### Exosome Injection

Eight‐week‐old male SD rats (n = 18) were randomized into three experimental groups and housed under controlled conditions. Following anesthesia induction with 2% pentobarbital sodium, a 22‐gauge needle was inserted 5 mm into the Co7/8 intervertebral space. The needle underwent 360° rotation followed by 30 s static retention. Subsequently, 5 µL of either PBS, vector‐Exos, or LAMP2A‐Exos was administered weekly for four weeks via microsyringe injection into the NP region. Upon completion of the 8‐week experimental period, X‐ray and MRI were conducted prior to tissue collection. Harvested IVDs underwent histological processing with H&E and SO staining for degeneration assessment.

### Small Animal In Vivo Imaging System

5 µm dil‐labeled exos (Sigma–Aldrich 42364) were administered to SD rats. Under 2% pentobarbital anesthesia, in vivo distribution was assessed using an IVIS Spectrum imaging system (PerkinElmer) at the injection site.

### Statistical Analysis

All statistical computations were performed using GraphPad Prism analytical software (v8.0). For pairwise comparisons between independent groups, parametric analysis employed the two‐tailed unpaired Student's t‐test when data exhibited normal distribution, while non‐parametric comparisons utilized the Mann‐Whitney U test for non‐normally distributed datasets. Multi‐group analyses involving three or more experimental conditions with a Gaussian distribution were conducted via two‐way ANOVA, followed by Tukey's multiple comparison test for pairwise contrasts. Statistical significance was defined as *p* < 0.05 for all analyses.

## Conflict of Interest

The authors declare no conflict of interest.

## Author Contributions

Z.W., Z.J., C.H., and S.Y. contributed equally to this work. Z.W. wrote the original draft and developed the methodology. Z.J. and C.H. performed investigations. S.Y. and S.J. performed the visualization. C.W., K.G., and J.L. conducted formal statistical analyses. S.X. curated data and managed databases. C.W. supervised project administration. X.W. performed conceptualization. C.W. and X.W. acquired funding. All authors read and approved the final manuscript.

## Supporting information



Supporting Information

## Data Availability

The data that support the findings of this study are available from the corresponding author upon reasonable request.
