# Supplementary material for: Acetylation Regulates ACSL4 Degradation Through Chaperone‐Mediated Autophagy to Alleviate Intervertebral Disc Degeneration
Source: Adv Sci (Weinh). 2025 Nov 21;13(8):e16015. doi: 10.1002/advs.202516015 (PMC12884747; doi:10.1002/advs.202516015)
Supplement: Supplementary file 1 — Supporting Information [file ADVS-13-e16015-s001.docx]

**Supplementary Information for**

**Acetylation regulates ACSL4 degradation through chaperone-mediated autophagy to alleviate intervertebral disc degeneration**

Zhouwei Wu^#,1,2,3,4^, Zhichen Jiang^#,1,2,3,4^, Chenglong Hong^#,1,2,3,4^, Shu Yang^#1,2,3,4^, Shuqing Jin^1,2,3,4^, Chenyu Wu^1,2,3,4^, Kaijie Guo^1,2,3,4^, Jiang Liu^1,2,3,4^, Shaobo Xu^1,2,3,4^, Chenggui Wang^1,2,3,4*^, Xiangyang Wang^1,2,3,4*^

**Affiliation**

1.Department of Orthopaedics, The Second Affiliated Hospital and Yuying Children's Hospital of Wenzhou Medical University, Wenzhou, 325027, China.

2.Key Laboratory of Orthopaedics of Zhejiang Province, Wenzhou, 325027, China.

3.Zhejiang Engineering Research Center for Innovation and Application of Intelligent Prevention and Treatment of Scoliosis in Children and Adolescents, Wenzhou, 325027, China

4.Zhejiang-Hong Kong Joint Laboratory for Precision Diagnosis and Treatment of Spinal Disorders, Wenzhou, 325027, China.

#These authors contributed equally to this work.

*Corresponding Authors: Chenggui Wang, Xiangyang Wang.

E-mail: wangchenggui@wmu.edu.cn (Chenggui Wang); xiangyangwang@wmu.edu.cn (Xiangyang Wang)

**
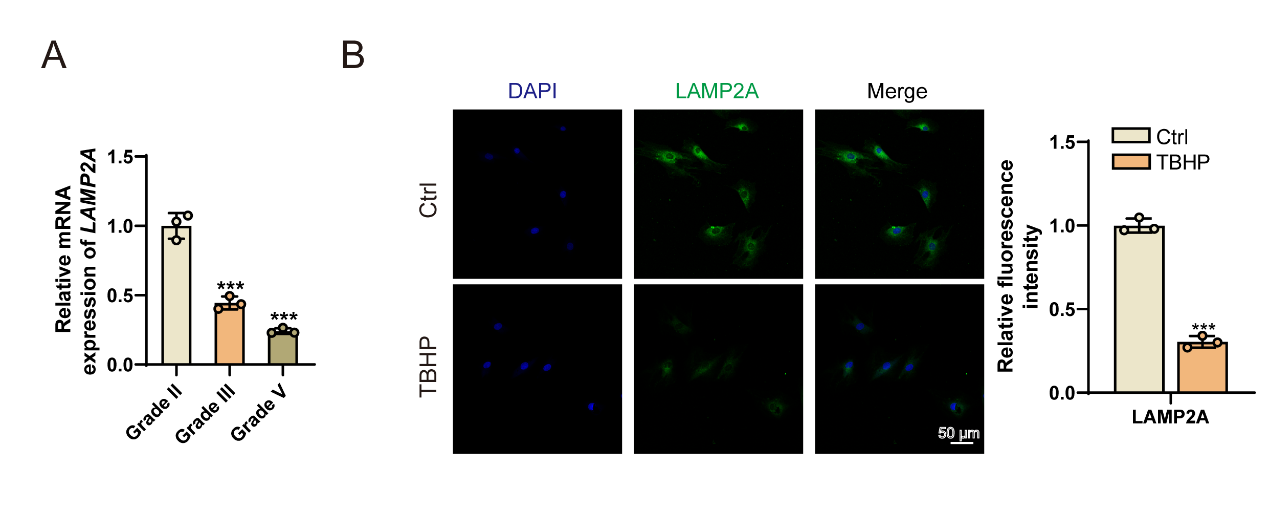
**

**Figure S1.** The expression level of LAMP2A in HsNPCs. (**A**) qPCR results demonstrated decreased *LAMP2A* expression in degenerative NP tissues. (n = 3). ***p < 0.001. (**B**)The IF confirmed the downregulation of LAMP2A in TBHP-stimulated HsNPCs. ***p < 0.001.


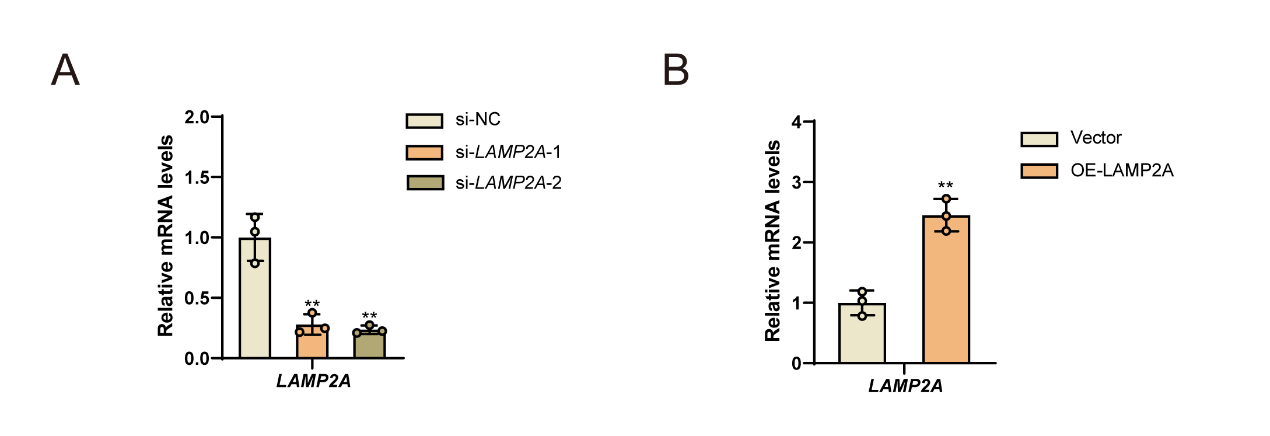


**Figure S2.** Verification of the efficiency of *LAMP2A* knockdown or overexpression. (**A**) *LAMP2A* mRNA expression levels by qRT-PCR in HsNPCs after *LAMP2A* knockdown (n = 3). **p < 0.01. (**B**) *LAMP2A* mRNA expression levels by qRT-PCR in HsNPCs after *LAMP2A* overexpression (n = 3). **p < 0.01.

**
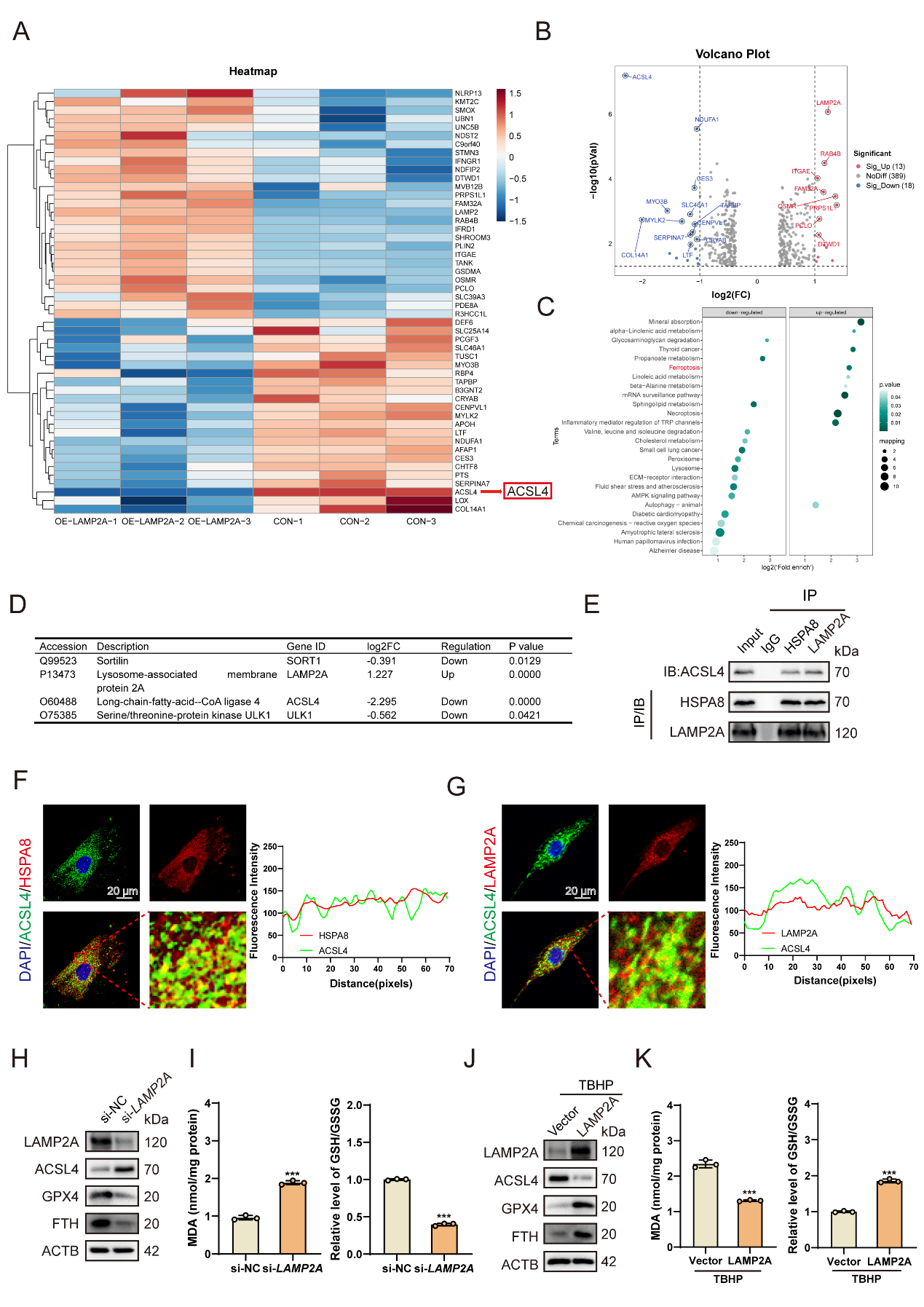
**

**Figure S3.** ACSL4 represents a potential substrate of CMA. (**A**) Differentially expressed genes in a heatmap. (**B**) Differentially expressed genes in a volcano map. (**C**) KEGG analysis. (**D**) Verify alterations in established CMA substrates within the proteomics data. (**E**) Co-IP confirmed the interaction between ACSL4, HSPA8 and LAMP2A in HsNPCs. (**F**) The interaction between HSPA8 and ACSL4 was confirmed by IF colocalization experiments. (**G**) The interaction between LAMP2A and ACSL4 was confirmed by IF colocalization experiments. (**H**) Protein expression levels of LAMP2A, ACSL4, GPX4, and FTH in HsNPCs with *LAMP2A* knockdown. (**I**) Analysis of intracellular malondialdehyde (MDA) levels and the glutathione/oxidized glutathione (GSH/GSSG) ratio in HsNPCs with *LAMP2A* knockdown. (**J**) Protein expression levels of LAMP2A, ACSL4, GPX4, and FTH in HsNPCs with *LAMP2A* overexpression. (**K**) Analysis of intracellular MDA levels and the GSH/GSSG ratio in HsNPCs with *LAMP2A* overexpression.


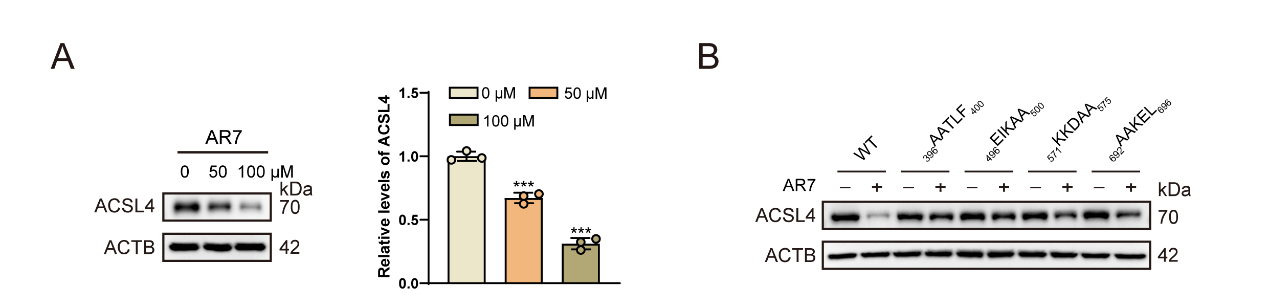


**Figure S4.** AR7 promotes the degradation of ACSL4. (**A**) Representative western blots of ACSL4 levels in HsNPCs treated with different concentrations of AR7 (0, 50, and 100 μM) for 24 h. (n = 3). ***p < 0.001. (**B**) The ACSL4 mutant is more resistant to AR7-induced degradation than the wild-type ACSL4.

**
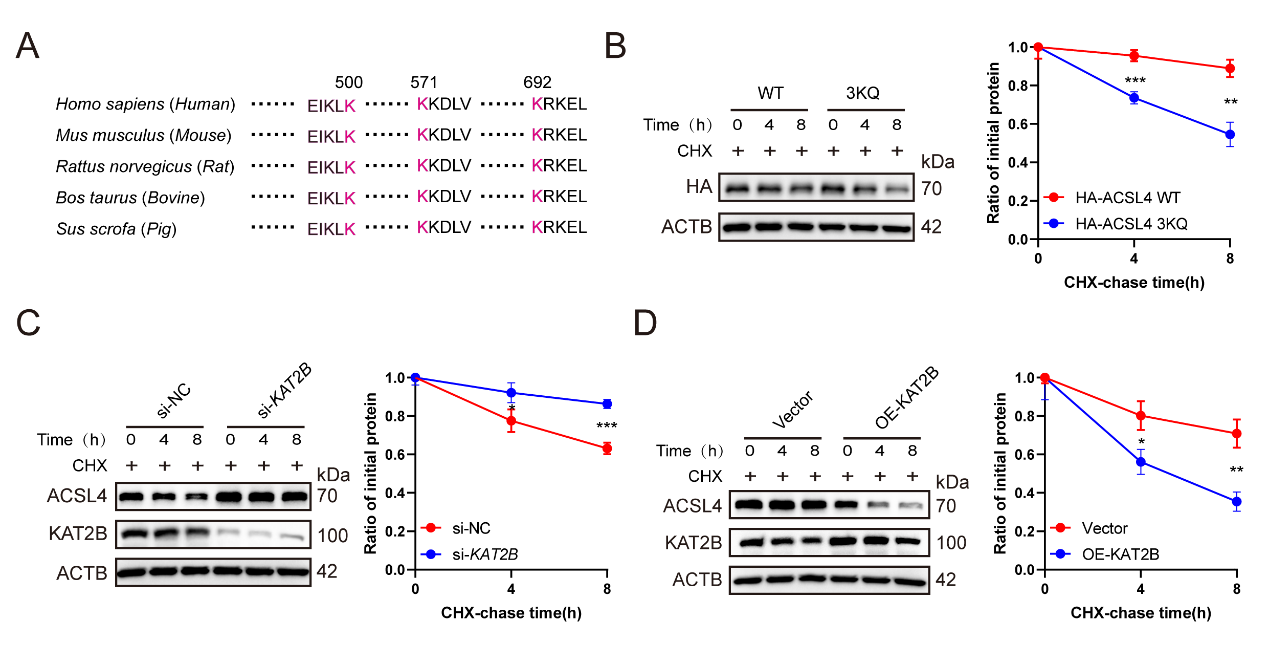
**

**Figure S5.**Assess amino acid sequence conservation across species. (**A**) Multiple-sequence alignment of ACSL4 amino acid sequences across species. Purple shading indicates conserved residues K500, K571, and K692. (**B**) The ACSL4-3KQ mutant is more susceptible to degradation than the WT protein. (**C**) *KAT2B* knockdown extended the half-life of ACSL4 protein. **p < 0.01, ***p < 0.001. (**D**) *KAT2B* overexpression shortened the half-life of ACSL4 protein. (n = 3). *p < 0.05, **p < 0.01.


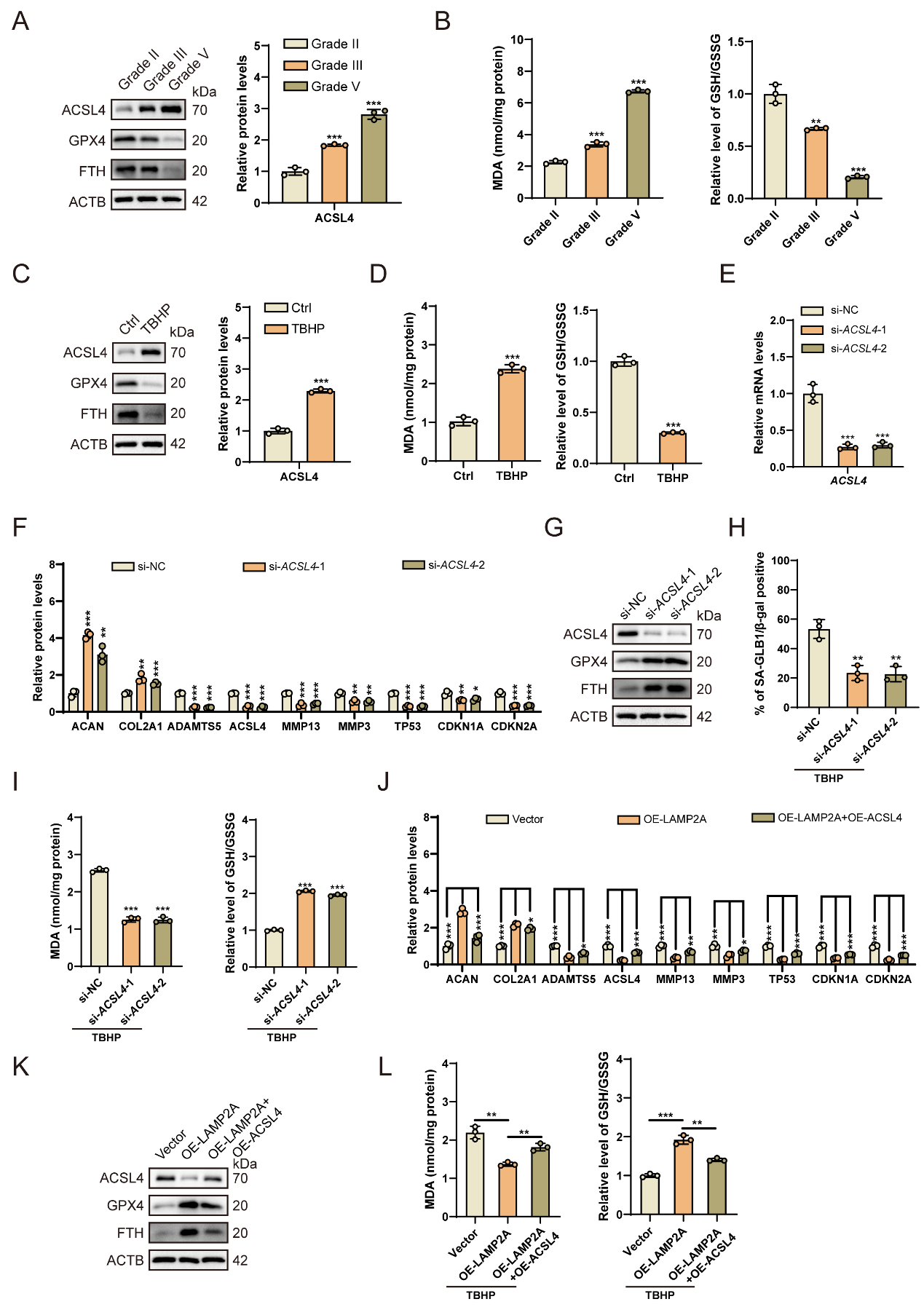


**Figure S6.** Intervertebral disc degeneration is associated with increased ferroptosis. (**A**) Representative western blots showing expression of ACSL4, GPX4, and FTH in human NP tissues. (n = 3). ***p < 0.001. (**B**) Analysis of intracellular MDA levels and the GSH/GSSG ratio in human NP tissues. (n = 3). **p < 0.01, ***p < 0.001. (**C**) Representative western blots showing expression of ACSL4, GPX4, and FTH in HsNPCs after TBHP treatment. (n = 3). ***p < 0.001. (**D**) Analysis of intracellular MDA levels and the GSH/GSSG ratio in HsNPCs after TBHP treatment. (n = 3). ***p < 0.001. (**E**) *ACSL4* mRNA expression levels by qRT-PCR in HsNPCs after *ACSL4* knockdown (n = 3). **p < 0.01. (**F**) Quantification of the western blot in (Figure S7A). (n = 3). *p < 0.05, **p < 0.01, ***p < 0.001. (**G**) Western blot was used to detect the protein levels of ACSL4, GPX4, and FTH in HsNPCs after *ACSL4* siRNAs treatment. (n = 3). (**H**) Quantitative analysis of SA-GLB1/β-gal staining in (Figure S7B). (**I**) Analysis of intracellular MDA levels and the GSH/GSSG ratio in HsNPCs after *ACSL4* siRNAs treatment. (n = 3). ***p < 0.001. (**J**) Quantification of the western blot in (Figure S7G). (n = 3). *p < 0.05, **p < 0.01, ***p < 0.001. (**K**) Western blot was used to detect the protein levels of ACSL4, GPX4, and FTH in HsNPCs when LAMP2A and ACSL4 were co-overexpressed. (n = 3). (**L**) Analysis of intracellular MDA levels and the GSH/GSSG ratio in HsNPCs when LAMP2A and ACSL4 were co-overexpressed. (n = 3). **p < 0.01, ***p < 0.001.

**
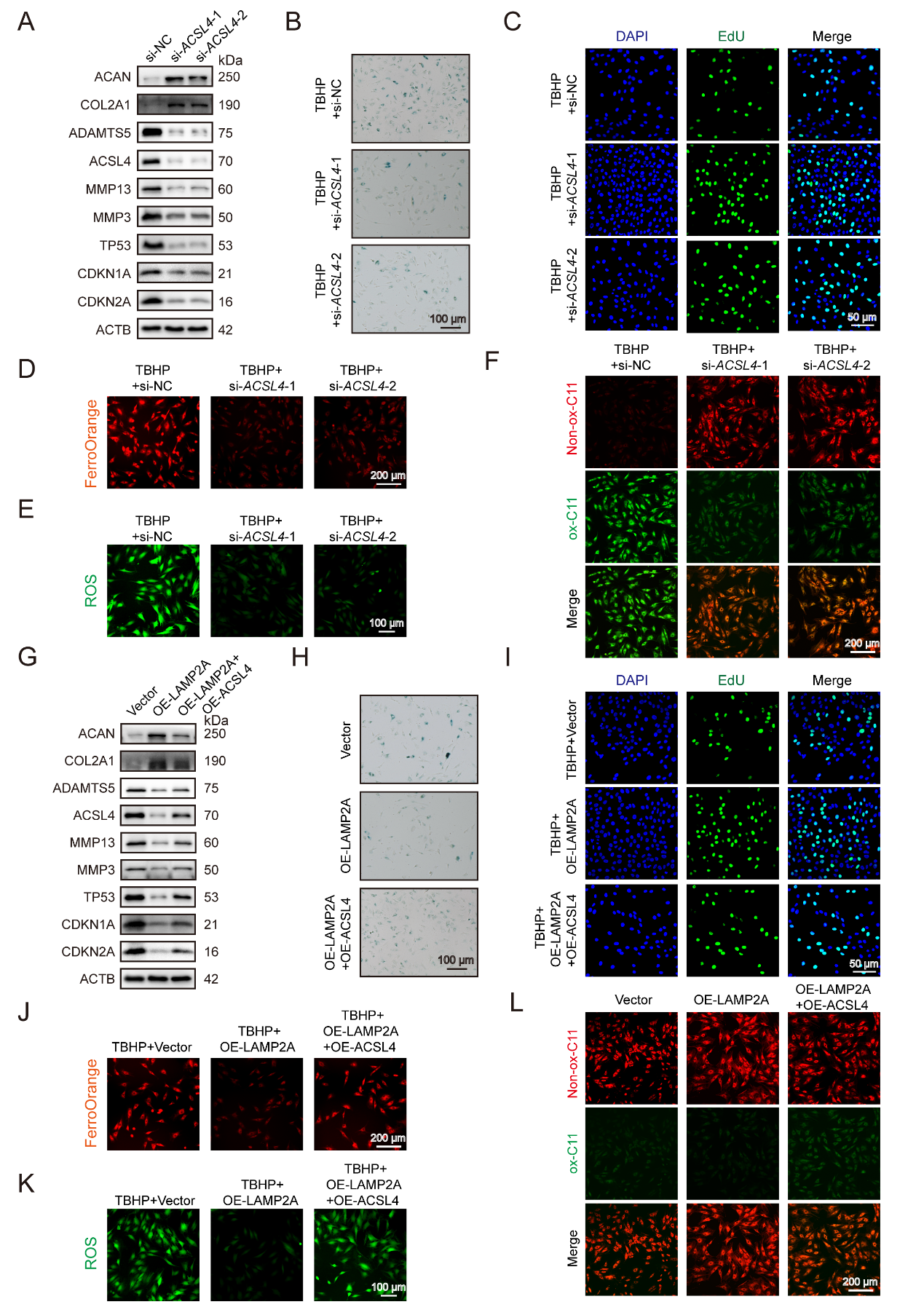
**

**Figure S7.** ACSL4 accumulation triggers ferroptosis-induced HsNPC senescence. (**A**) Western blot was used to detect the protein levels of ACAN, COL2A1, ACSL4, ADAMTS5,MMP13, MMP3, TP53, CDKN1A, and CDKN2A in HsNPCs after *ACSL4* siRNAs treatment. (n = 3). (**B**) Representative images of SA-GLB1/β-gal staining in HsNPCs when *ACSL4* was knockdown. (**C**) *ACSL4* knockdown significantly enhanced HsNPCs proliferation as quantified by EdU assay. (n = 3). (**D**) Determination of Fe^2+^ concentration using FerroOrange probe. (**E**) Measure the level of ROS using the DCFH-DA probe. (**F**) Measurement of Lipid Peroxidation Level using BODIPY 581/591 C11 Probe. (**G**) Western blot was used to detect the protein levels of ACAN, COL2A1, ACSL4, ADAMTS5,MMP13, MMP3, TP53, CDKN1A, and CDKN2A in HsNPCs when LAMP2A and ACSL4 were co-overexpressed. (n = 3). (**H**) Representative images of SA-GLB1/β-gal staining in HsNPCs when LAMP2A and ACSL4 were co-overexpressed. (n = 3). (**I**) The proliferation ability of HsNPCs was detected by the EdU assay when ACSL4 and LAMP2A were co-overexpressed. (**J**) Determination of Fe^2+^ concentration using FerroOrange probe. (**K**) Measure the level of ROS using the DCFH-DA probe. (**L**) Measurement of Lipid Peroxidation Level using BODIPY 581/591 C11 Probe.

**
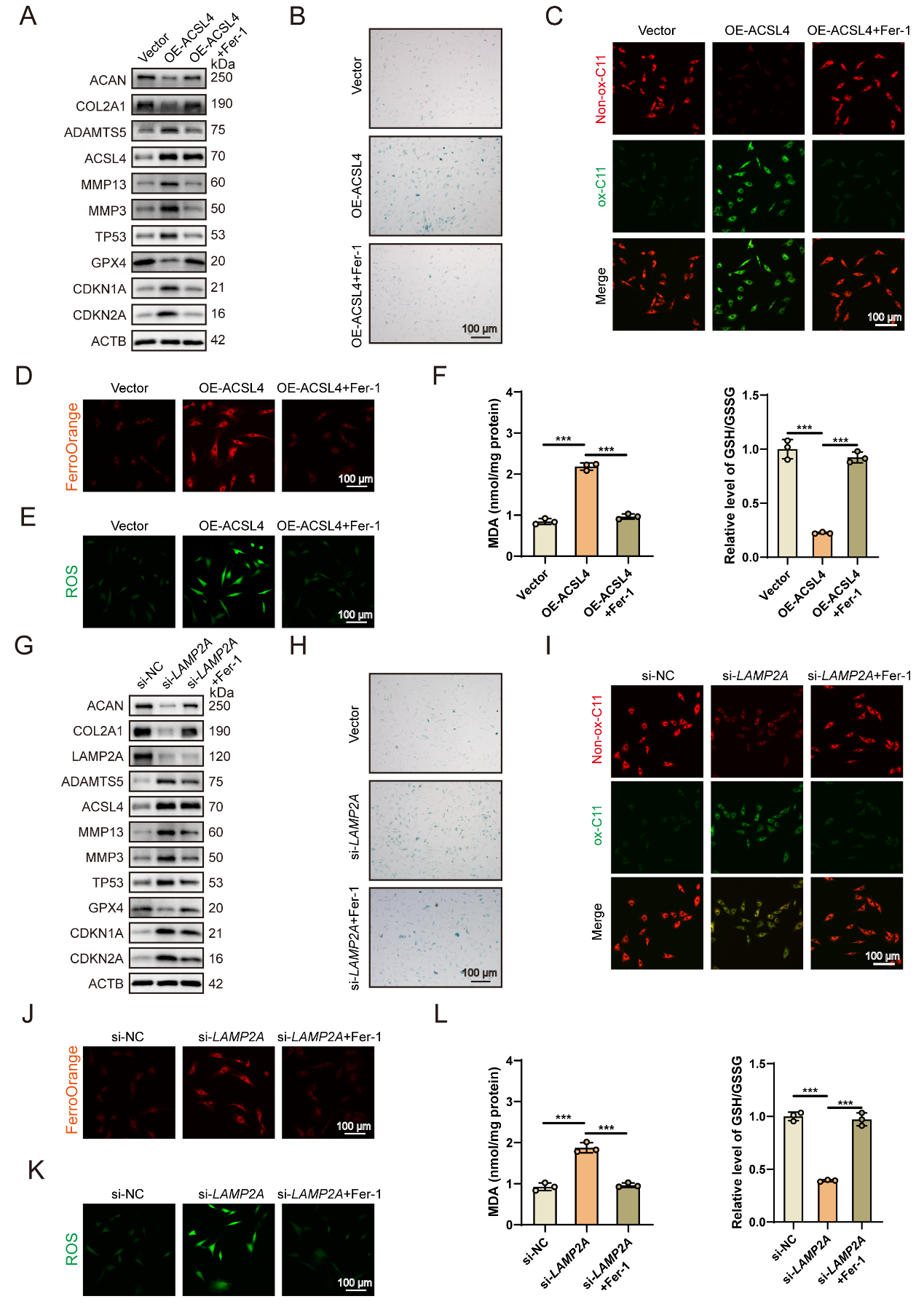
**

**Figure S8.** The Roles of ACSL4 and LAMP2A in Cellular Functions and Ferroptosis. (**A**) Western blot was used to detect the protein levels of ACAN, COL2A1, ACSL4, ADAMTS5,MMP13, MMP3, TP53, GPX4, CDKN1A, and CDKN2A in HsNPCs with *ACSL4* overexpression and Fer-1 treatment. (n = 3). (**B**) Representative images of SA-GLB1/β-gal staining in HsNPCs with *ACSL4* overexpression and Fer-1 treatment. (n = 3). (**C**) Measurement of Lipid Peroxidation Level using BODIPY 581/591 C11 Probe. (**D**) Determination of Fe^2+^ concentration using FerroOrange probe. (**E**) Measure the level of ROS using the DCFH-DA probe. (**F**) Analysis of intracellular MDA levels and the GSH/GSSG ratio in HsNPCs with *ACSL4* overexpression and Fer-1 treatment. (n = 3). ***p < 0.001. (**G**) Western blot was used to detect the protein levels of ACAN, COL2A1, ACSL4, ADAMTS5,MMP13, MMP3, TP53, GPX4, CDKN1A, and CDKN2A in HsNPCs with *LAMP2A* knockdown and Fer-1 treatment. (n = 3). (**H**) Representative images of SA-GLB1/β-gal staining in HsNPCs with *LAMP2A* knockdown and Fer-1 treatment. (n = 3). (**I**) Measurement of Lipid Peroxidation Level using BODIPY 581/591 C11 Probe. (**J**) Determination of Fe^2+^ concentration using FerroOrange probe. (**K**) Measure the level of ROS using the DCFH-DA probe. (**L**) Analysis of intracellular MDA levels and the GSH/GSSG ratio in HsNPCs with *LAMP2A* knockdown and Fer-1 treatment. (n = 3). ***p < 0.001.


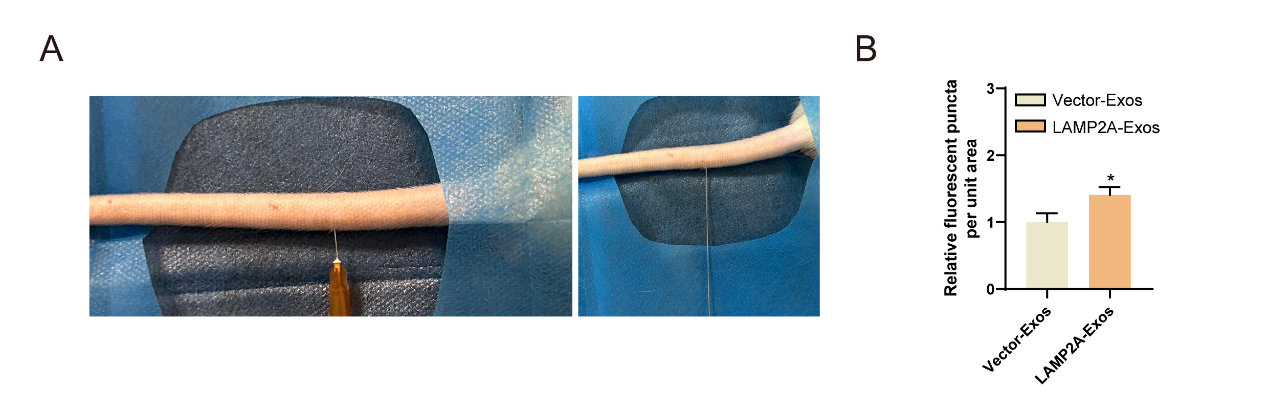


**Figure S9.** Schematic diagram of animal modeling. (**A**) The left panel depicts percutaneous needle injury methodology to establish IVDD models, while the right panel shows the microinjection apparatus employed for AAV and Exos transduction. (**B**) Quantification of exosome uptake was expressed as fluorescent puncta per unit area (puncta/μm²). (n = 3). *p < 0.05.

**Supplementary Tables**

**Table S1 Sequences of siRNAs**

| Human *LAMP2A* siRNA#1 | GCAGUGCAGAUGACGACAATT |
| --- | --- |
| Human *LAMP2A* siRNA#2 | GAGUACUUAUUCUAGUGUUTT |
| Human *KAT2B* siRNA | GCAGATACCAAACAAGTTTA |
| Human *ACSL4* siRNA#1 | GCAAAGAAGCAGUAGUUCATT |
| Human *ACSL4* siRNA#2 | GCAGTAGTTCATGGGCTAA |

**Table S2 Antibodies used in this study**

| anti-CDKN2A antibody | Abclonal (A0262) |
| --- | --- |
| annti-CDKN1A antibody | Proteintech (10355-1-AP) |
| anti-TP53 antibody | Proteintech (10442-1-AP) |
| anti-COL2A1 antibody | Santa Cruz Biotechnology (sc-52658) |
| anti–ACAN antibody | Abcam (ab315486) |
| anti–ADAMTS5 antibody | Abcam (ab41037) |
| anti–MMP13 antibody | Abcam (ab39012) |
| anti-MMP3 antibody | Santa Cruz Biotechnology (sc-21732) |
| anti-HSPA8 antibody | Proteintech (10654-1-AP) |
| anti-Flag antibody | Proteintech (20543-1-AP) |
| anti-His antibody | Proteintech (66005-1-Ig) |
| anti-GST antibody | Proteintech (10000-0-AP) |
| anti–MYC antibody | Proteintech (60003-2-Ig) |
| anti–HA antibody | Proteintech (51064-2-AP) |
| anti-LAMP2A antibody | Abcam (ab125068) |
| anti-LAMP1 antibody | Proteintech (21997-1-AP) |
| anti-ACSL4 antibody | Santa Cruz Biotechnology (sc-365230) |
| anti-acetylated-lysine antibody | Cell Signaling Technology (9441) |
| anti-SQSTM1/p62 antibody | Abcam (ab109012) |
| anti-TSG101 antibody | Huabio (ET1701-59) |
| anti-CD9 antibody | Huabio (HA721533) |
| Anti-CD81 antibody | Proteintech (27855-1-AP) |
| Anti-CANX antibody | Proteintech (10427-2-AP) |
| Anti-ACTB antibody | Proteintech (66009-1-Ig) |

**Table S3 Primer sequences employed in this study**

| ACTB  (human) | F: AGAGCTACGAGCTGCCTGAC  R: AGCACTGTGTTGGCGTACAG |
| --- | --- |
| LAMP2A  (human) | F: TGTCTGGAGCATTTCAGATAAA  R: ATGGGCACAAGGAAGTTG |
| ACSL4  (human) | F: AAGTAGACCAACGCCTTCA  R: CAGTCCAGGTATTCTTTCACA |

**Table S4 IP-MS analysis**

| Gene | # Unique Peptides | Coverage [%] | Abundance | Sum PEP Score | Sequest HT Score |
| --- | --- | --- | --- | --- | --- |
| ACAT1 | 11 | 38 | 269334979.5 | 93.416 | 53.79 |
| ACAT2 | 10 | 42 | 201761380.3 | 73.154 | 43.42 |
| NAA15 | 17 | 18 | 156483424 | 58.706 | 37.56 |
| HDAC1 | 7 | 30 | 133063442.5 | 52.695 | 30.23 |
| KAT2B | 14 | 18 | 177343564.5 | 52.086 | 30.46 |
| HAT1 | 6 | 22 | 82603642.5 | 40.987 | 22.02 |
| HDAC2 | 5 | 23 | 45923935.5 | 40.19 | 24.46 |
| NAT10 | 8 | 10 | 21156060.75 | 28.021 | 11.45 |
| NAA50 | 6 | 34 | 123625909 | 23.707 | 14.56 |
| NAA25 | 5 | 5 | 21677189.5 | 19.904 | 10.55 |
| DLAT | 5 | 7 | 97105984.63 | 19.024 | 14.02 |
| GNPNAT1 | 4 | 28 | 22871596.25 | 15.773 | 5.2 |
| NAA16 | 3 | 3 | 34943029 | 11.654 | 7.93 |
| NAA10 | 3 | 12 | 39645902 | 10.574 | 7.2 |
| KAT2A | 3 | 3 | 28731925 | 8.597 | 7.21 |
